# Molecular Pharmacological Characterization of Dicentrine Isolated from *Stephania venosa* in Human Lymphoma Cells

**DOI:** 10.3390/ijms27156974

**Published:** 2026-08-03

**Authors:** Aroonchai Saiai, Sirinya Moakmamern, Lapamas Rueankum, Wenxian Yin, Singkome Tima, Siriporn Okonogi, Sawitree Chiampanichayakul, Songyot Anuchapreeda

**Affiliations:** 1 Department of Chemistry, Faculty of Science, Chiang Mai University, Chiang Mai 50200, Thailand; aroonchai.s@cmu.ac.th; 2 Department of Medical Technology, Faculty of Associated Medical Sciences, Chiang Mai University, Chiang Mai 50200, Thailand; sirinya_moakm@cmu.ac.th (S.M.); lapamas.ru@cmu.ac.th (L.R.); yinwenxian@swmu.edu.cn (W.Y.); singkome.tima@cmu.ac.th (S.T.); sawitree.chiampa@cmu.ac.th (S.C.); 3 Center of Excellence in Pharmaceutical Nanotechnology, Chiang Mai University, Chiang Mai 50200, Thailand; okng2000@gmail.com; 4 Cancer Research Unit of Associated Medical Sciences (AMS CRU), Faculty of Associated Medical Sciences, Chiang Mai University, Chiang Mai 50200, Thailand; 5 Department of Pharmaceutical Sciences, Faculty of Pharmacy, Chiang Mai University, Chiang Mai 50200, Thailand

**Keywords:** dicentrine, *Stephania venosa*, cancer, lymphoma, c-Myc, apoptosis, PI3K/Akt signaling

## Abstract

Lymphoma remains a major hematological malignancy associated with treatment resistance and systemic toxicity, highlighting the need for novel anticancer agents derived from natural products. In this study, dicentrine (**5**), an aporphine alkaloid isolated from *Stephania venosa*, was investigated for its anti-lymphoma activity in Raji and Ramos cells. Among the isolated compounds, dicentrine (**5**) exhibited the strongest cytotoxic activity, with IC_50_ values of 9.03 ± 0.53 and 5.16 ± 0.44 µg/mL in Raji and Ramos cells, respectively, while demonstrating favorable selectivity toward lymphoma cells relative to peripheral blood mononuclear cells (PBMCs). Dicentrine (**5**) significantly suppressed c-Myc and phosphorylated c-Myc expression, reduced lymphoma cell proliferation, and decreased total viable cell numbers in a dose-dependent manner. Cell cycle analysis revealed G0/G1 arrest in Raji cells and G2/M arrest in Ramos cells. Furthermore, dicentrine (**5**) induced apoptosis, as evidenced by increased Annexin V-positive populations and elevated cleaved caspase-3 expression. Molecular docking analysis demonstrated strong binding affinities of dicentrine (**5**) toward Akt, PI3K, caspase-3, and caspase-9, while network pharmacology identified AKT1 and the PI3K/Akt signaling pathway as potential targets associated with lymphoma suppression. Western blot analysis further demonstrated that dicentrine (**5**) significantly reduces total Akt protein expression. Overall, the present findings indicate that dicentrine (**5**) suppresses lymphoma progression by inhibiting cell proliferation and promoting apoptotic cell death, highlighting its potential as a promising natural therapeutic candidate for lymphoma.

## 1. Introduction

Lymphoma comprises a diverse group of malignancies originating from transformed lymphocytes that undergo uncontrolled expansion within the lymphoid tissues. It is broadly classified into Hodgkin lymphoma and non-Hodgkin lymphoma, with the latter comprising the majority of cases and encompassing diverse subtypes with distinct pathological and molecular characteristics [1,2,3]. Although current therapeutic approaches have substantially improved patient management, disease recurrence, drug resistance, and treatment-associated toxicity remain major clinical challenges. In particular, cytotoxic regimens often induce off-target toxicity in normal tissues and may compromise the quality of life of patients. Despite remarkable advances in targeted therapies and immunotherapies for lymphoma, including Bruton tyrosine kinase inhibitors, bispecific antibodies, antibody–drug conjugates, and chimeric antigen receptor (CAR)-T cell therapy, treatment resistance, disease relapse, and therapy-related toxicities remain major clinical challenges [4,5,6]. Consequently, the identification and development of novel therapeutic agents with improved efficacy, enhanced selectivity, and reduced toxicity continue to represent an important priority in lymphoma research.

Medicinal plants continue to represent an important reservoir of structurally diverse molecules for anticancer drug discovery. Numerous plant-derived bioactive molecules, such as alkaloids, flavonoids, and terpenoids, have demonstrated potent antiproliferative and pro-apoptotic effects against various cancer types. Among these, isoquinoline alkaloids have attracted increasing attention because of their diverse pharmacological activities, including anti-inflammatory, antioxidant, antimicrobial, and anticancer properties [7]. Previous pharmacological investigations have demonstrated that dicentrine exhibits multiple biological activities, including anti-inflammatory, antioxidant, and anticancer properties. [8,9]. However, its effects on lymphoma cells remain insufficiently characterized. Although dicentrine is a known aporphine alkaloid, its anti-lymphoma activity and underlying molecular mechanisms remain largely unknown. Therefore, the present study aimed to characterize the pharmacological effects of dicentrine isolated from *Stephania venosa* against human lymphoma cells and elucidate the molecular mechanisms responsible for its anti-lymphoma activity.

*Stephania venosa* (Blume) Spreng., a medicinal plant belonging to the family Menispermaceae, is traditionally used in several Asian countries, including Thailand, for the treatment of various disorders. Previous phytochemical investigations of *S. venosa* have identified numerous aporphine and isoquinoline alkaloids, including dicentrine, crebanine, tetrahydropalmatine, dehydrodicentrine, and dehydroisolaureline. These alkaloids have been reported to exhibit diverse pharmacological activities, including anti-inflammatory, antioxidant, antimicrobial, and anticancer effects. Among them, dicentrine has attracted increasing attention because of its antiproliferative and pro-apoptotic activities in several human cancer models, suggesting its potential as a promising anticancer candidate [10,11]. The ethanolic extract exhibited the most potent cytotoxic activity against human small lung cancer (NCI-H187) and human breast cancer (MCF-7) cells, with an IC_50_ values of 4.88 and 19.76 µg/mL, respectively. Among the identified constituents, dicentrine has emerged as a promising candidate owing to its reported ability to modulate cell survival signaling pathways and induce cancer cell death through apoptotic mechanism. A previous study demonstrated that dicentrine potentiates TNF-α-induced apoptosis and suppresses invasion in A549 lung adenocarcinoma cells. Moreover, dicentrine increases the activities of caspase-8, -9, -3, as well as poly (ADP-ribose) polymerase (PARP) cleavage, by upregulating the death-inducing signaling complex and downregulating the expression of antiapoptotic proteins, including cIAP2, cFLIP, and Bcl-XL. Furthermore, dicentrine significantly blocks TNF-α-activated TAK1, p38, JNK, and Akt signaling pathways, leading to reduced transcriptional activities of NF-κB and AP-1 [12]. The anticancer potential of dicentrine isolated from *S. venosa* in lymphoma has not been fully explored. These findings suggest that dicentrine may exert its anticancer effects through the modulation of cell survival and apoptosis-related signaling pathways. Since cell proliferation and programmed cell death are fundamental biological processes involved in tumor progression and therapeutic response, dysregulation of these processes is closely associated with cancer development. Uncontrolled proliferation is a hallmark of cancer, whereas the induction of cell death, particularly apoptosis, is a major mechanism underlying the efficacy of anticancer agents. Therefore, compounds capable of suppressing lymphoma cell growth and promoting cell death may represent a promising therapeutic strategy.

However, despite the promising pharmacological properties of dicentrine, its anti-lymphoma activity and underlying molecular mechanisms remain poorly understood. In the present study, five alkaloid compounds were extracted, purified, and structurally characterized from *Stephania venosa*, and their cytotoxic activities were systematically evaluated against human lymphoma cell lines, including Raji and Ramos cells, together with normal peripheral blood mononuclear cells (PBMCs) to assess anticancer selectivity. The most potent compound was subsequently investigated for its effects on lymphoma cell proliferation, cell cycle progression, and apoptosis induction. Furthermore, molecular docking and network pharmacology analyses were integrated to identify the potential signaling pathways and therapeutic targets associated with its anti-lymphoma activity. Collectively, this study provides new evidence supporting dicentrine (**5**) as a promising bioactive alkaloid targeting lymphoma cell survival and highlights its potential for further development as a natural product-derived therapeutic agent for lymphoma treatment.

## 2. Results

### 2.1. Yield of Extract

**Figure 1:**
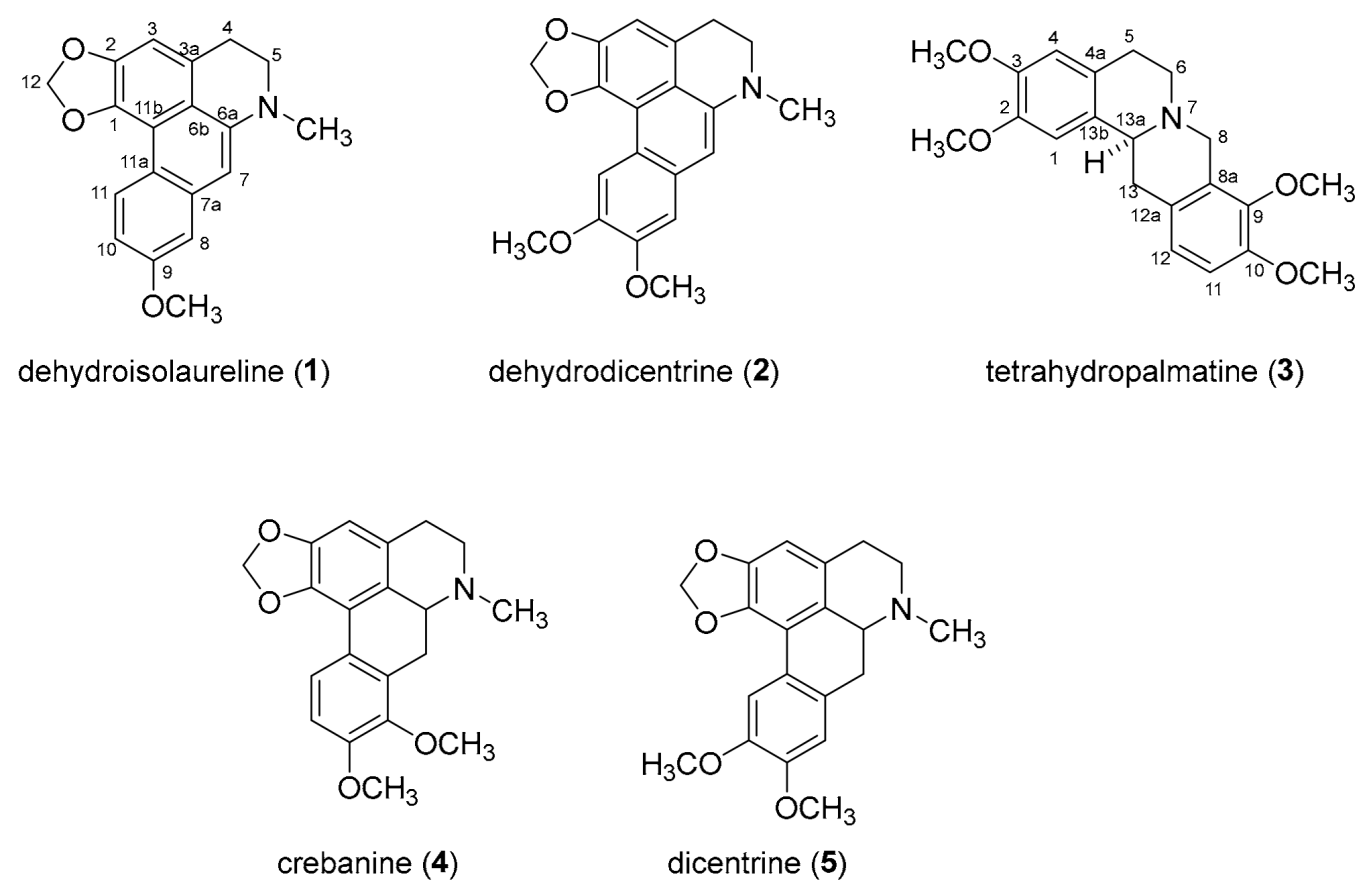
Chemical structure of isolated compounds (**1**–**5**) from tubers of *S. venosa*.

The dried and finely ground tubers of *S. venosa* (6.0 kg) were extracted using ethyl acetate, and methanol at room temperature. After removal of the solvents, the crude extracts were obtained as follows: 148.58 g (2,48%) of ethyl acetate extract and 61.89 g (1.03%) of methanol extract, respectively. A portion of ethyl acetate extract (11.97 g) was further separated using column chromatography, yielding four aporphine alkaloids—dehydroisolaureline (**1**) (12.2 mg, 0.0025% *w*/*w*), dehydrodicentrine (**2**) (365.0 mg, 0.0755% *w*/*w*), crebanine (**4**) (256.9 mg, 0.0531% *w*/*w*), and dicentrine (**5**) (756.8 mg, 0.1565% *w*/*w*)—as well as one protoberberine alkaloid, tetrahydropalmatine (**3**) (471.6 mg, 0.0976% *w*/*w*) with yields expressed relative to the dried plant material (Figure 1). The structures of these isolated compounds were identified using spectroscopic techniques and compared with previously reported data [9,13,14,15,16,17,18,19,20].

Overall, the EtOAc extract contained higher proportions of nonpolar alkaloids tetrahydropalmatine (**3**) and dicentrine (**5**), which are the major constituents in this fraction, in agreement with previous report [21], whereas the MeOH extract was enriched in more polar alkaloids, such as Palmatine and Jatrorrhizine, which are quaternary protoberberine-type alkaloids [22]. This compositional difference likely contributes to the stronger activity observed for the EtOAc extract.

### 2.2. Extraction and Isolation

The structures of isolated compounds **1**–**5** were all shown in Figure 1. After analysis of ^1^H and ^13^C NMR spectra (Figure S1–S10) and comparison with known compounds reported in the literature, compounds **1**–**5** were identified as dehydroisolaureline (**1**), dehydrodicentrine (**2**), tetrahydropalmatine (**3**), crebanine (**4**), and dicentrine (**5**). The complete ^1^H and ^13^C NMR spectroscopic data for the five alkaloids (**1**–**5**) are presented.

Dehydroisolaureline (**1**) Brownish gum ^1^H-NMR (CDCl_3_) ^1^H NMR (500 MHz, Chloroform-*d*) δ 8.71 (d, *J* = 9.0 Hz, 1H, H-11), 6.95 (d, *J* = 2.7 Hz, 1H, H-8), 6.85 (dd, *J* = 9.0, 2.7Hz, 1H, H-10), 6.78 (s, 1H, H-3), 6.41 (s, 1H, H-7), 6.09 (s, 2H, H-12), 3.84 (s, 3H, 9-OCH_3_), 3.26 (t, *J* = 6.1 Hz, 2H, H-5), 3.12 (t, *J* = 6.1 Hz, 2H, H-4), 2.98 (s, 3H, N-CH_3_) [13,14].

^13^C NMR (125 MHz, CDCl_3_) δ 158.53 (C-9), 145.09 (C-2), 144.21 (C-6a), 141.38 (C-1), 135.86 (C-7a), 128.46 (C-11), 127.50 (C-3a), 118.22 (C-6b), 117.47 (C-11a), 117.45 (C-11b), 112.17 (C-8), 106.55 (C-10), 106.50 (C-3), 100.84 (C-7), 100.67 (C-12), 55.20 (9-OCH_3_), 50.57 (C-5), 40.34 (N-CH_3_), 30.84 (C-4) [13].

Dehydrodicentrine (**2**) ^1^H NMR (500 MHz, CDCl_3_) δ 8.38 (s, 1H, H-11), 7.02 (s, 1H, H-3), 6.87 (s, 1H, H-8), 6.49 (s, 1H, H-7), 6.18 (s, 2H, H-12), 4.01 (s, 3H, 9-OCH_3_), 4.00 (s, 3H, 10-OCH_3_), 3.32 (t, *J* = 6.0 Hz, 2H, H-5), 3.21 (t, *J* = 6.0 Hz, 2H, H-4), 3.04 (s, 3H, N-CH_3_) [16].

^13^C NMR (126 MHz, CDCl_3_) δ 149.35 (C-9), 145.86 (C-10), 144.62 (C-2), 142.79 (C-1), 141.14 (C-6a), 129.38 (C-7a), 127.59 (C-3a), 118.46 (C-6b), 117.20 (C-11a), 116.98 (C-11b), 108.41 (C-11), 106.66 (C-8), 106.31 (C-3), 100.79 (C-7), 100.69 (C-12), 56.02 (9-OCH_3_), 55.70 (10-OCH_3_), 50.66 (C-5), 40.37 (N-CH_3_), 31.06 (C-4) [15].

Tetrahydropalmatine (**3**) ^1^H NMR (500 MHz, CDCl_3_) δ 6.87 (d, *J* = 8.3 Hz, 1H, H-12), 6.78 (d, *J* = 8.4 Hz, 1H, H-11), 6.73 (s, 1H, H-1), 6.61 (s, 1H, H-4), 4.24 (d, *J* = 15.8 Hz, 1H, H-8), 3.88 (s, 3H, 3-OCH_3_), 3.86 (s, 3H, 2-OCH_3_), 3.85 (s, 3H, 9-OCH_3_), 3.84 (s, 3H, 10-OCH_3_), 3.54 (d, *J* = 16.0 Hz, 1H, H-8), 3.53 (d, *J* = 3.3 Hz, 1H, H-13a), 3.26 (dd, *J* = 15.8, 3.7 Hz, 1H, H-13_ax_), 3.23–3.16 (m, 1H, H-6), 3.16–3.09 (m, 1H, H-5), 2.83 (dd, *J* = 15.7, 11.4 Hz, 1H, H-13_eq_), 2.70–2.60 (m, 4H, H-5, H-6) [16,17,18].

^13^C NMR (126 MHz, CDCl_3_) δ 150.29 (C-10), 147.50 (C-3),147.45 (C-2) 145.07 (C-9), 129.67 (C-13b), 128.64 (C-12a), 127.72 (C-8a), 126.79 (C-4a), 123.89 (C-12), 111.36 (C-4), 110.97 (C-11), 108.61 (C-1), 60.19 (9-OCH_3_), 59.33 (C-13a), 56.09 (2-OCH_3_), 55.88 (3-OCH_3_), 55.86 (10-OCH_3_), 54.00 (C-8), 51.52 (C-6), 36.31 (C-13), 29.09 (C-5) [16,17,18].

Crebanine (**4**) ^1^H NMR (500 MHz, CDCl_3_) δ 7.79 (d, *J* = 8.6 Hz, 1H, H-11), 6.86 (d, *J* = 8.6 Hz, 1H, H-10), 6.51 (s, 1H, H-3), 6.05 (s, 1H, H-12), 5.89 (s, 1H, H-12), 3.89 (s, 3H, 9-OCH_3_), 3.80 (s, 3H, 8-OCH_3_), 3.66 (dd, *J* = 14.7, 4.3 Hz, 1H, H-7), 3.15–3.02 (m, 3H, H-4, H-5, H-6a), 2.61 (dd, *J* = 15.9, 3.4 Hz, 1H, H-4), 2.58 (s, 3H, N-CH_3_), 2.52 (td, *J* = 11.9, 11.0, 3.1 Hz, 1H, H-5), 2.30 (t, *J* = 14.1 Hz, 1H, H-7) [16,17,18].

^13^C NMR (126 MHz, CDCl_3_) δ 152.04 (C-8), 146.61 (C-2), 145.84 (C-9), 142.09 (C-1), 129.72 (C-7a), 126.48 (C-3a), 126.41 (C-6b), 124.61 (C-11a), 123.13 (C-11), 116.52 (C-11b), 110.28 (C-10), 106.84 (C-3), 100.63 (C-12), 61.86 (C-6a), 60.72 (8-OCH_3_), 55.77 (9-OCH_3_), 53.56 (C-5), 43.85 (N-CH_3_), 29.03 (C-4), 26.83 (C-7) [16,17,18].

Dicentrine (**5**) ^1^H NMR (500 MHz, CDCl_3_) δ 7.66 (s, 1H, H-11), 6.77 (s, 1H, H-8), 6.51 (s, 1H, H-3), 6.07 (d, *J* = 1.5 Hz, 1H, H-12), 5.92 (d, *J* = 1.5 Hz, 1H, H-12), 3.92 (s, 3H), 3.91 (s, 3H), 3.16–3.00 (m, 4H, H-4,H-5, H-6a, H-7), 2.67–2.59 (m, 2H, H-4, H-7), 2.54 (s, 3H, N-CH_3_), 2.50 (td, *J* = 11.9, 3.8 Hz, 1H, H-5) [9,19,20].

^13^C NMR (125 MHz, CDCl_3_) δ 148.27 (C-10), 147.70 (C-9), 146.63 (C-1), 141.80 (C-2), 128.38 (C-7a), 126.66 (C-3a), 126.46 (C-6b), 123.57 (C-11a), 116.62 (C-11b), 111.29 (C-8), 110.54 (C-11), 106.79 (C-3), 100.63 (C-12), 62.42 (C-6a), 56.12 (10-OCH_3_), 55.90 (9-OCH_3_), 53.60 (C-5), 43.95 (N-CH_3_), 34.25 (C-7), 29.23 (C-4) [9,20].

### 2.3. Cytotoxic Effects of S. venosa Crude Extracts on Raji and Ramos Lymphoma Cells and PBMCs

This study assessed the cytotoxic effects of methanol (MeOH) crude extract and ethyl acetate (EtOAc) crude extract against Raji and Ramos lymphoma cell lines, together with normal PBMCs. The EtOAc crude extract showed stronger cytotoxic activity in both cell lines, with IC_50_ values of 24.76 ± 1.70 and 14.89 ± 1.36 µg/mL in Raji and Ramos cells, respectively (Table 1). To evaluate the chemotherapeutic potential of the crude extract, the selectivity index (SI) was determined. Both extracts exhibited SI values greater than 1, indicating preferential cytotoxicity toward lymphoma cells compared with normal PBMCs. Notably, the EtOAc crude extract demonstrated favorable SI values of 2.66 in Raji cells and 4.42 in Ramos cells and was therefore selected for further separated by column chromatography.

### 2.4. Purification and Cytotoxic Screening of Active Compounds from S. venosa Against Raji and Ramos Lymphoma Cells, and PBMCs

The cytotoxic effects of the five isolated compounds from the EtOAc crude extract–dehydroisolaureline (**1**), dehydrodicentrine (**2**), tetrahydropalmatine (**3**), crebanine (**4**) and dicentrine (**5**)—were evaluated against Raji and Ramos lymphoma cell lines, together with normal PBMCs. Dicentrine (**5**) displayed the strongest cytotoxic activity among all isolated compounds, with IC_50_ values of 9.03 ± 0.53 µg/mL in Raji cells and 5.16 ± 0.44 µg/mL in Ramos cells, while the remaining compounds exhibited varying levels of activity. Moreover, dicentrine (**5**) exhibited minimal toxicity toward normal PBMCs, with IC_50_ values of 41.44 ± 5.33 µg/mL. The SI values of dicentrine (**5**) were greater than 1, indicating preferential cytotoxicity toward lymphoma cells than PBMCs, with SI values of 4.59 in Raji cells and 8.03 in Ramos cells (Table 2). Therefore, subsequent experiments were conducted to further investigate the antiproliferative and cell death-inducing effects of EtOAc crude extract and dicentrine (**5**).

### 2.5. Effects of EtOAc Crude Extract and Dicentrine (***5***) on c-Myc and Phosphorylated c-Myc Protein Expression in Raji and Ramos Lymphoma Cells

**Figure 2:**
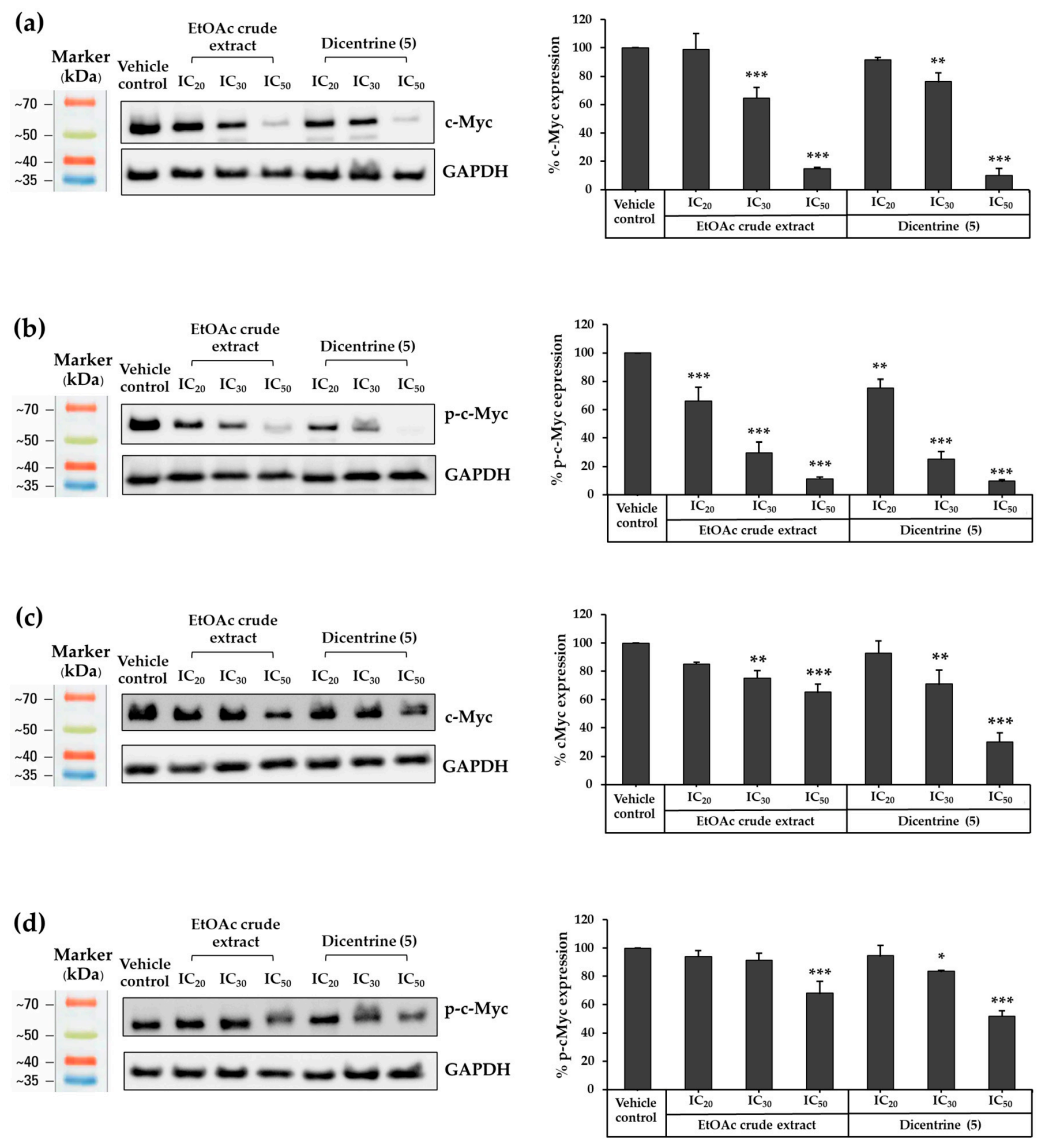
Effects of EtOAc crude extract and dicentrine (**5**) on c-Myc and p-c-Myc protein expression in Raji and Ramos cells. Cells were treated at IC_20_, IC_30_, and IC_50_ concentrations for 48 h, and protein expression levels were assessed by Western blotting. (**a**) c-Myc expression in Raji cells. (**b**) p-c-Myc expression in Raji cells. (**c**) c-Myc expression in Ramos cells. (**d**) p-c-Myc expression in Ramos cells. Each bar represents the mean ± SD of three independent experiments. Protein levels were quantified by densitometric analysis and normalized to GAPDH, which served as the loading control. Asterisks (*) indicate statistically significant differences compared with the vehicle control (* *p* < 0.05, ** *p* < 0.01, *** *p* < 0.001).

c-Myc is an important transcription factor in lymphoma, regulating key cellular processes including cell growth, proliferation, and programmed cell death. It serves as a central regulator of cell cycle progression and is widely recognized as a biomarker in lymphoma [23]. In this study, both c-Myc and its phosphorylated (activated) from, p-c-Myc, were assessed as indicators of lymphoma cell proliferation. Raji and Ramos cells were treated with the EtOAc crude extract and dicentrine (**5**) at concentrations corresponding to IC_20_, IC_30_, and IC_50_ for 48 h, and the expression levels of c-Myc and p-c-Myc were subsequently analyzed by Western blotting. In Raji cells, the concentrations of the EtOAc crude extract corresponding to IC_20_, IC_30_, and IC_50_ were 11.71, 16.74, and 24.76 µg/mL, respectively, whereas those used in Ramos cells were 7.10, 9.68, and 14.89 µg/mL, respectively. For dicentrine (**5**), the concentrations used in Raji cells were 3.30, 4.78, and 9.03 µg/mL, respectively, while the corresponding concentrations in Ramos cells were 3.10, 3.81, and 5.16 µg/mL, respectively. Both treatments significantly suppressed c-Myc and p-c-Myc protein expression in Raji and Ramos cells in a dose-dependent manner. In Raji cells, treatment with the EtOAc crude extract for 48 h significantly reduced c-Myc protein expression by 35.31 and 85.15% of the vehicle control at IC_30_ and IC_50_ concentrations, respectively. Dicentrine (**5**) produced a comparable effect, significantly reducing c-Myc levels by 23.68 and 89.81% of control at IC_30_ and IC_50_ concentrations, respectively (Figure 2a). Both treatments also significantly suppressed p-c-Myc expressions. After 48 h of exposure, the EtOAc crude extract reduced p-c-Myc expression by 33.88, 70.22, and 88.66% of control at IC_20_, IC_30_, and IC_50_ concentrations, respectively, whereas dicentrine (**5**) decreased p-c-Myc levels by 24.57, 74.66, and 90.30% under the same conditions (Figure 2b). In Ramos cells, however, the suppressive effects were less pronounced. Treatment with the EtOAc crude extract reduced c-Myc expression by 24.81 and 34.55% of control at IC_30_ and IC_50_ concentrations, respectively, while dicentrine (**5**) decreased c-Myc levels by 29.10 and 69.97% of control at IC_30_ and IC_50_ concentrations, respectively (Figure 2c). The effects on p-c-Myc expression were relatively modest, with significant suppression observed only at higher concentrations. Specifically, the EtOAc crude extract reduced p-c-Myc expression by 39.06% of control at IC_50_ concentration, whereas dicentrine (**5**) decreased p-c-Myc levels by 16.56 and 48.06% of control at IC_30_ and IC_50_ concentrations, respectively (Figure 2d). Collectively, these findings indicate that both the EtOAc crude extract and dicentrine (**5**) effectively suppress c-Myc signaling in lymphoma cells, with the inhibitory effect being more pronounced in Raji cells. Notably, dicentrine (**5**) demonstrated stronger activity than the EtOAc crude extract, highlighting its potential as a more potent modulator of c-Myc–driven pathways.

### 2.6. Effects of EtOAc Crude Extract and Dicentrine (***5***) on the Proliferation Rate of Raji and Ramos Lymphoma Cells

**Figure 3:**
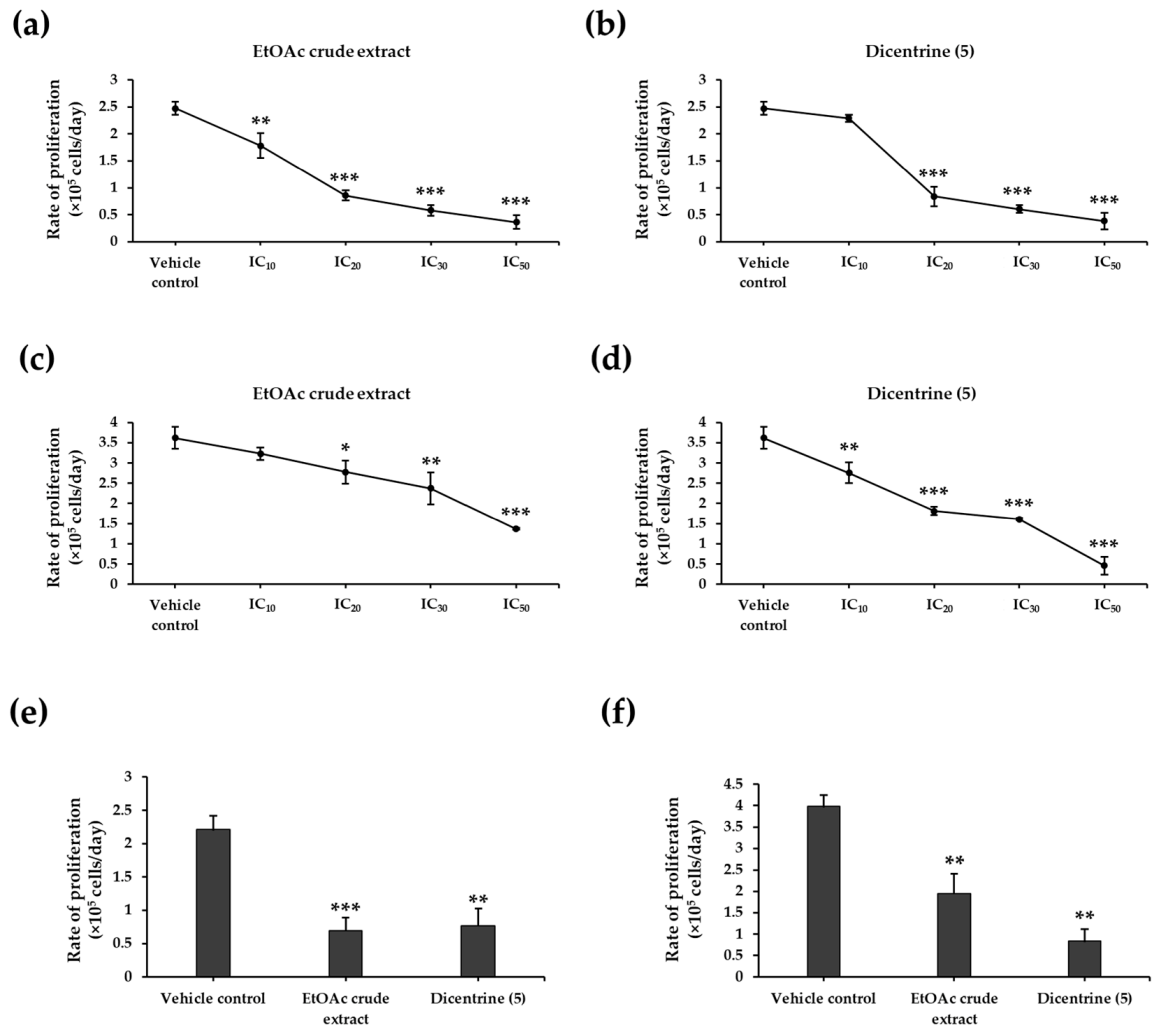
Effects of EtOAc crude extract and dicentrine (**5**) on proliferation rate of Raji and Ramos lymphoma cells. Cells were treated with IC_10_, IC_20_, IC_30_, and IC_50_ concentrations for 48 h: (**a**) Raji cells treated with EtOAc crude extract, (**b**) Raji cells treated with dicentrine (**5**), (**c**) Ramos cells treated with EtOAc crude extract, and (**d**) Ramos cells treated with dicentrine (**5**). Time-course experiments were performed at IC_20_ concentrations for 12, 24, 48, and 72 h in (**e**) Raji and (**f**) Ramos cells. Cell numbers were determined by trypan blue exclusion assay. Data represent mean ± SD of three independent experiments. Asterisks indicate statistically significant differences compared with the vehicle control (* *p* < 0.05, ** *p* < 0.01, *** *p* < 0.001).

c-Myc is a critical transcription factor that regulates cell growth and proliferation in lymphoma cells. Suppression of c-Myc expression markedly reduces cell proliferation in Raji and Ramos cells. To confirm these effects, cell proliferation was quantified using the trypan blue exclusion assay. Both the EtOAc crude extract and dicentrine (**5**) significantly inhibited proliferation at IC_20_, IC_30_ and IC_50_ concentrations after 48 h (Figure 3a–d). At lower concentrations, EtOAc crude extract at IC_10_ (7.85 µg/mL) significantly reduced proliferation in Raji cells (Figure 3a), whereas dicentrine (**5**) at IC_10_ concentration (0.57 µg/mL) significantly decreased proliferation in Ramos cells (Figure 3d). Time-course analysis performed at 12, 24, 48, and 72 h further demonstrated proliferation patterns consistent with dose-dependent responses. Both the EtOAc crude extract and dicentrine (**5**) consistently suppressed lymphoma cell proliferation over time in both cell lines, with dicentrine (**5**) exhibiting stronger antiproliferative activity. At 72 h, dicentrine (**5**) reduced cell proliferation by 65.31% in Raji cells and 79.07% in Ramos cells. Similarly, the EtOAc crude extract significantly inhibited proliferation by 68.62 and 51.07% in Raji and Ramos cells, respectively (Figure 3e,f).

### 2.7. Effect of EtOAc Crude Extract and Dicentrine (***5***) on Akt Protein Expression

**Figure 4:**
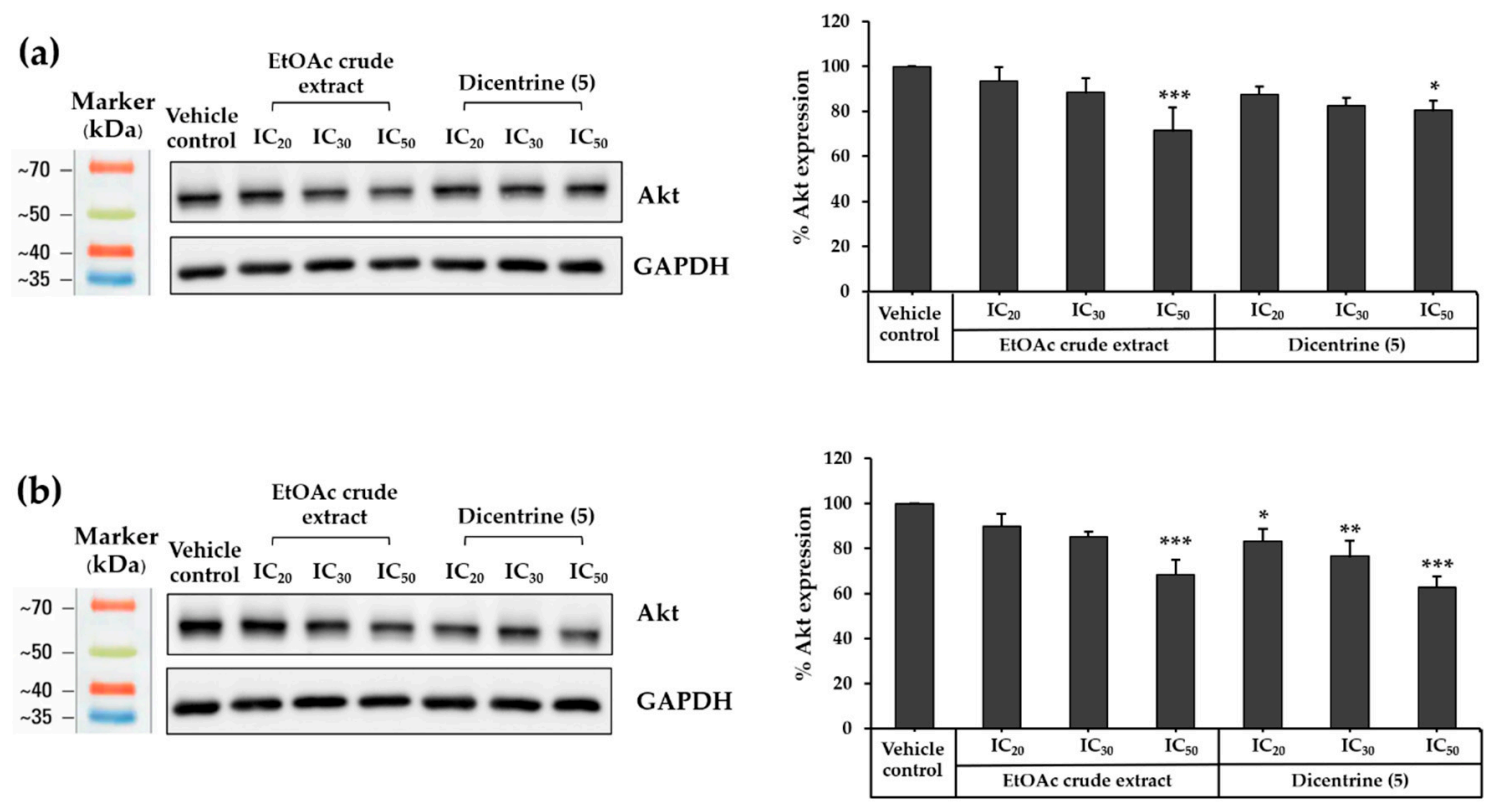
Effects of EtOAc crude extract and dicentrine (**5**) on Akt expression in Raji and Ramos lymphoma cells. Cells were treated with IC_20_, IC_30_, and IC_50_ concentrations for 48 h, and protein expression levels were assessed by Western blotting. (**a**) Akt expression in Raji cells. (**b**) Akt expression in Ramos cells. Data represent mean ± SD of three independent experiments. Asterisks indicate statistically significant differences compared with the vehicle control (* *p* < 0.05, ** *p* < 0.01, *** *p* < 0.001).

To further investigate the molecular mechanisms underlying the anti-lymphoma activity of the EtOAc crude extract and dicentrine (**5**), total Akt protein expression was examined by Western blot analysis. Raji and Ramos cells were treated with the EtOAc crude extract or dicentrine (**5**) at IC_20_, IC_30_, and IC_50_ concentrations for 48 h. Both the EtOAc crude extract and dicentrine (**5**) reduced total Akt protein expression in the two lymphoma cell lines, with a more pronounced effect observed in Ramos cells. In Raji cells, a significant reduction in Akt protein expression was observed only at the IC_50_ concentration. The EtOAc crude extract reduced Akt protein expression by 28.50%, whereas dicentrine (**5**) reduced Akt expression by 19.42%, relative to the vehicle control (Figure 4a). In contrast, Ramos cells exhibited a dose-dependent reduction in Akt protein expression. The EtOAc crude extract reduced Akt expression by 31.39% at the IC_50_ concentration, whereas dicentrine (**5**) reduced Akt protein expression by 16.69%, 23.22%, and 37.05% at the IC_20_, IC_30_, and IC_50_ concentrations, respectively, relative to the vehicle control (Figure 4b). These findings indicate that both the EtOAc crude extract and dicentrine (**5**) suppress total Akt protein expression in lymphoma cells, suggesting the involvement of Akt in their anti-lymphoma activity.

### 2.8. Effect of EtOAc Crude Extract and Dicentrine (***5***) on Total Cell Number in Raji and Ramos Cells

**Figure 5:**
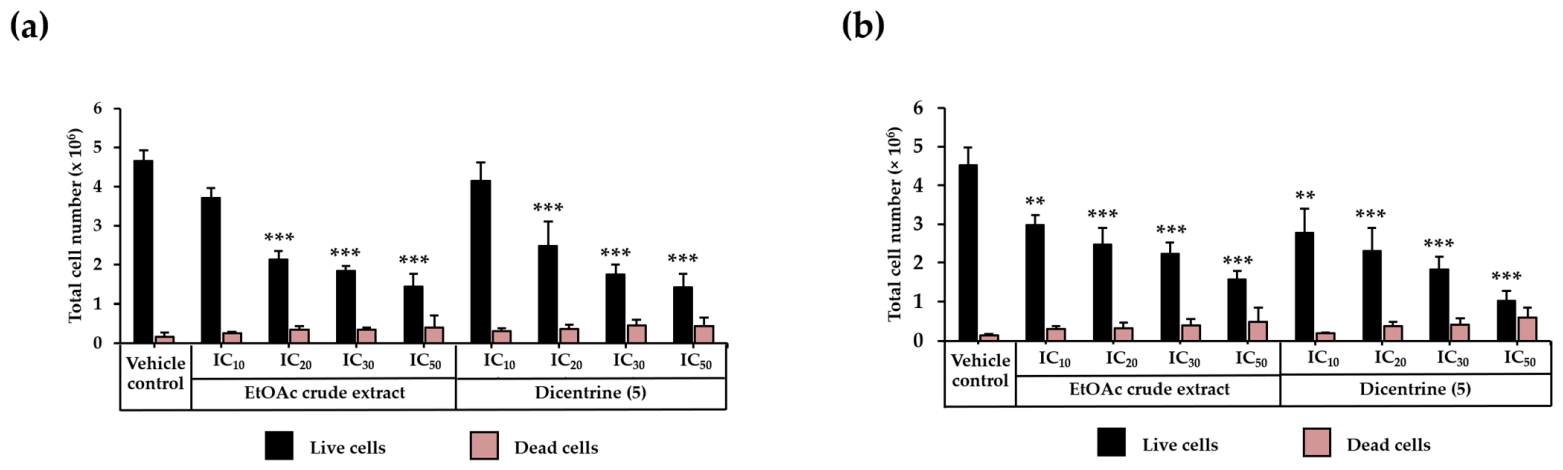
Effects of EtOAc crude extract and dicentrine (**5**) on total cell number in Raji and Ramos lymphoma cells. Cells were treated with IC_10_, IC_20_, IC_30_, and IC_50_ concentrations for 48 h, and total cell numbers were determined by trypan blue exclusion assay. (**a**) Raji cells and (**b**) Ramos cells. Data represent mean ± SD of three independent experiments. Asterisks indicate statistically significant differences compared with the vehicle control (** *p* < 0.01, *** *p* < 0.001).

Treatment with the EtOAc crude extract and dicentrine (**5**) led to a marked reduction in total cell numbers without a proportional increase in cell death. Cell viability, assessed by trypan blue exclusion after 48 h of treatment at IC_10_, IC_20_, IC_30_, and IC_50_ concentrations, revealed a clear dose-dependent decline in viable cells across both lymphoma cell lines. In Raji cells, increasing concentrations of the EtOAc crude extract reduced the number of viable cells by 20.07, 54.30, 60.55, and 68.94% at IC_10_, IC_20_, IC_30_, and IC_50_ concentrations, respectively. Significant reductions were observed at IC_20_, IC_30_, and IC_50_ concentrations (Figure 4a). Similarly, dicentrine (**5**) decreased viable cell numbers by 10.84, 46.63, 62.50, and 69.39% at the corresponding concentrations, with statistically significant reductions detected at IC_20_, IC_30_, and IC_50_ concentrations (Figure 5a). A comparable dose-dependent trend was observed in Ramos cells, where the EtOAc crude extract significantly reduced viable cell numbers by 33.93, 45.36, 50.37, and 65.24%, whereas dicentrine (**5**) further decreased viable cell numbers by 38.54, 49.07, 59.50, and 77.32% following treatment with increasing concentrations (Figure 5b). At IC_50_ concentrations, treatment with the EtOAc crude extract and dicentrine (**5**) produced only a relatively small proportion of non-viable cells in Raji cells, accounting for 21.95 and 23.50% of total cell population, respectively (Figure 4a). In Ramos cells, non-viable cells comprised 23.92 and 36.49% of the total cell population following exposure to the EtOAc crude extract and dicentrine (**5**), respectively (Figure 5b). These findings suggest that both treatments primarily suppress lymphoma cell proliferation prior to extensive induction of cell death.

### 2.9. Effect of EtOAc Crude Extract and Dicentrine (***5***) on Cell Cycle Distribution

**Figure 6:**
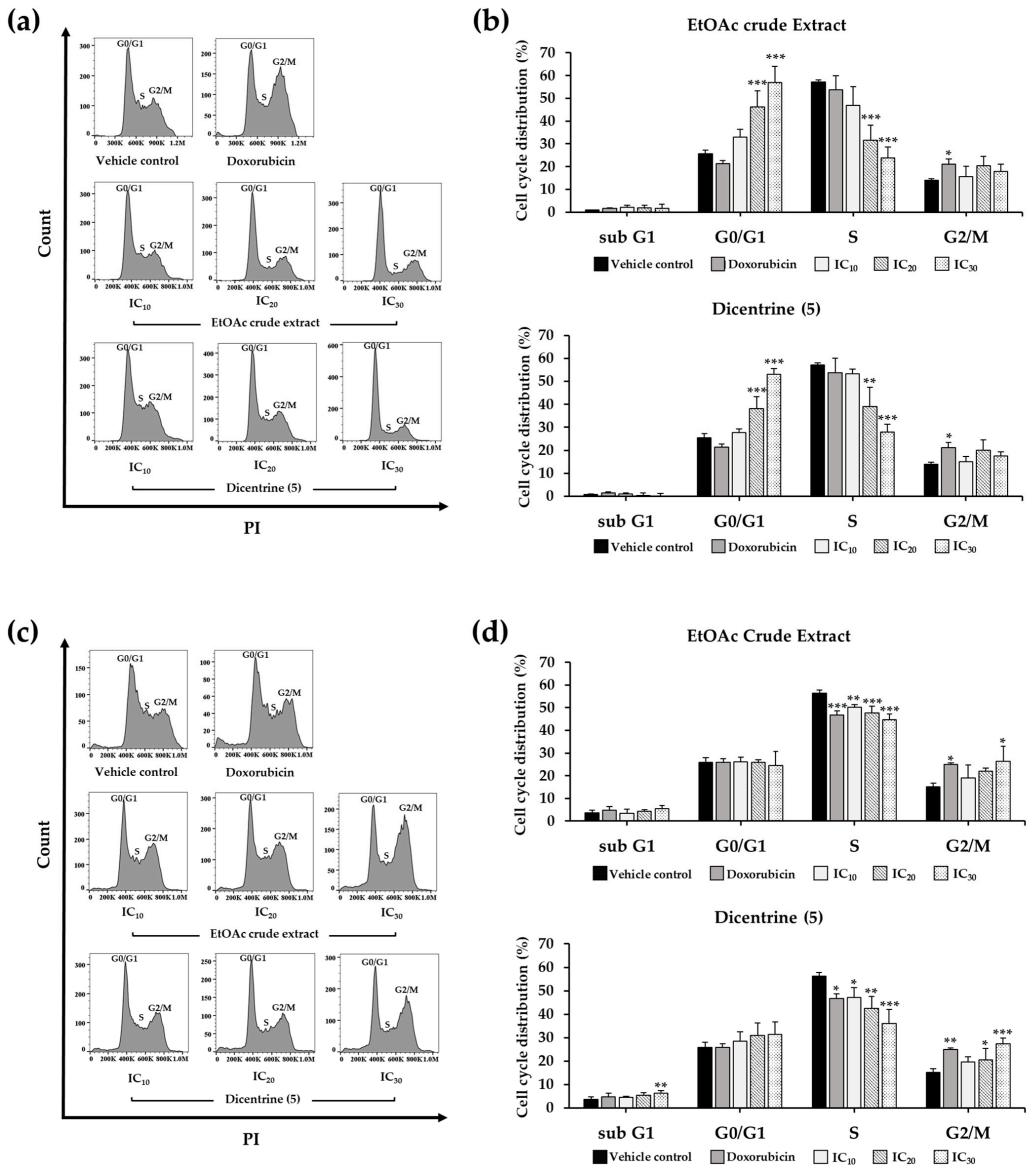
Effects of EtOAc crude extract and dicentrine (**5**) on cell cycle distribution in Raji and Ramos lymphoma cells. Cells were treated with IC_10_, IC_20_, and IC_30_ concentrations for 48 h, and cell cycle distribution was analyzed by propidium iodide (PI) staining and flow cytometry. (**a**) Representative histograms and (**b**) quantitative analysis of Raji cells; (**c**) representative histograms and (**d**) quantitative analysis of Ramos cells. Doxorubicin served as positive control. Data represent the mean ± SD of three independent experiments. Asterisks indicate statistically significant differences compared with the vehicle control (* *p* < 0.05, ** *p* < 0.01, *** *p* < 0.001).

To further validate the antiproliferative effects of the EtOAc crude extract and dicentrine (**5**), cell cycle distribution was analyzed in Raji and Ramos cells. Raji and Ramos cells, seeded at densities of 1 × 10^5^ cells/mL and 1.25 × 10^5^ cells/mL, respectively, were treated with each compound at concentrations corresponding to their IC_10_, IC_20_, and IC_30_ values. Doxorubicin was used as the positive control. Following a 48 h of treatment, cell cycle progression was evaluated by propidium iodide (PI) staining and flow cytometric analysis. As expected, doxorubicin induced G2/M phase arrest in both lymphoma cell lines. In Raji cells, treatment with EtOAc crude extract and dicentrine (**5**) significantly increased the proportion of cells in the G0/G1 phase at IC_20_ and IC_30_ concentrations compared with the vehicle control, indicating G0/G1 phase arrest (Figure 6a,b). In contrast, Ramos cells exhibited a different response pattern. Treatment with the EtOAc crude extract at IC_30_ concentration and dicentrine (**5**) at IC_20_ and IC_30_ concentrations significantly induced G2/M phase accumulation, resembling the cell cycle distribution pattern observed with doxorubicin (Figure 6c,d). Furthermore, dicentrine (**5**) at high concentrations significantly increased the sub-G1 population in Ramos cells, suggesting induction of apoptotic cell death. Collectively, these findings demonstrate that the EtOAc crude extract and dicentrine (**5**) isolated from *S. venosa* exert antiproliferative effects in lymphoma cells through induction of G0/G1 arrest in Raji cells and G2/M arrest in Ramos cell.

### 2.10. Effects of EtOAc Crude Extract and Dicentrine (***5***) on Cleaved Caspase-3 Protein Expression in Raji and Ramos Lymphoma Cells

**Figure 7:**
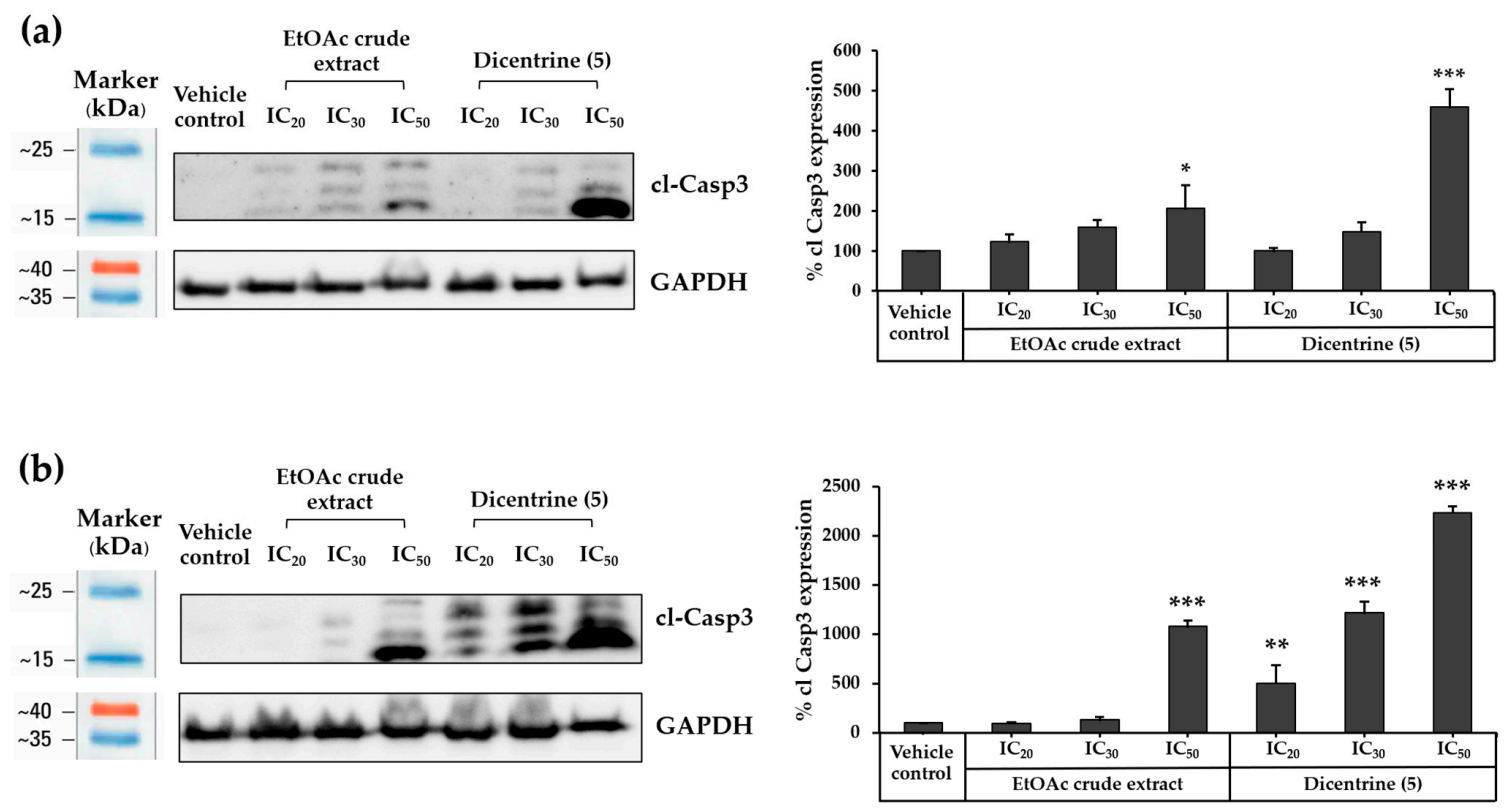
Effects of EtOAc crude extract and dicentrine (**5**) on cl-Casp3 protein expression in Raji and Ramos lymphoma cell lines. Cells were treated with IC_20_, IC_30_, and IC_50_ concentrations for 48 h, and cl-Casp3 protein levels were analyzed by Western blotting. (**a**) Raji cells and (**b**) Ramos cells. Protein expression levels were quantified by densitometric analysis and normalized to GAPDH. Data represent the mean ± SD of three independent experiments. Asterisks indicate statistically significant differences compared with the vehicle control (* *p* < 0.05, ** *p* < 0.01, *** *p* < 0.001).

Activation of cleaved caspase-3 (cl-Casp3) is a central hallmark of apoptosis, functioning as a key executioner protease that dismantles cellular structures and drives programmed cell death. Molecular docking analysis previously identified Casp3 among the top five strongest binding targets of dicentrine (**5**), underscoring its relevance in apoptotic regulation. In this study, the pro-apoptotic effects of the EtOAc crude extract and dicentrine (**5**) were examined in Raji and Ramos lymphoma cells by assessing cl-Casp3 protein expression following 48 h of treatment at IC_20_, IC_30_, and IC_50_ concentrations. Both of them induced cl-Casp3 expression in a dose-dependent manner, with Ramos cells exhibiting the greatest sensitivity. In Raji cells, EtOAc crude extract treatment produced a 2.05-fold increase, while dicentrine (**5**) induced a 4.59-fold increase at IC_50_ compared to vehicle control (Figure 7a) However, in Ramos cells, dicentrine (**5**) significantly elevated cl-Casp3 levels by 5.04-, 12.15-, and 22.34-fold at IC_20_, IC_30_, and IC_50_, respectively, whereas EtOAc crude extract produced a 10.77-fold increase at IC_50_ concentration compared to vehicle control (Figure 7b) Notably, dicentrine (**5**) consistently induced stronger cl-Casp3 upregulation than the EtOAc crude extract, in line with its more potent pro-apoptotic activity. Collectively, these findings confirm that both treatments activate the intrinsic Casp3-mediated apoptotic pathway in lymphoma cells, with dicentrine (**5**) demonstrating superior efficacy.

### 2.11. Effect of EtOAc Crude Extract and Dicentrine (***5***) on Lymphoma Cell Apoptosis

**Figure 8:**
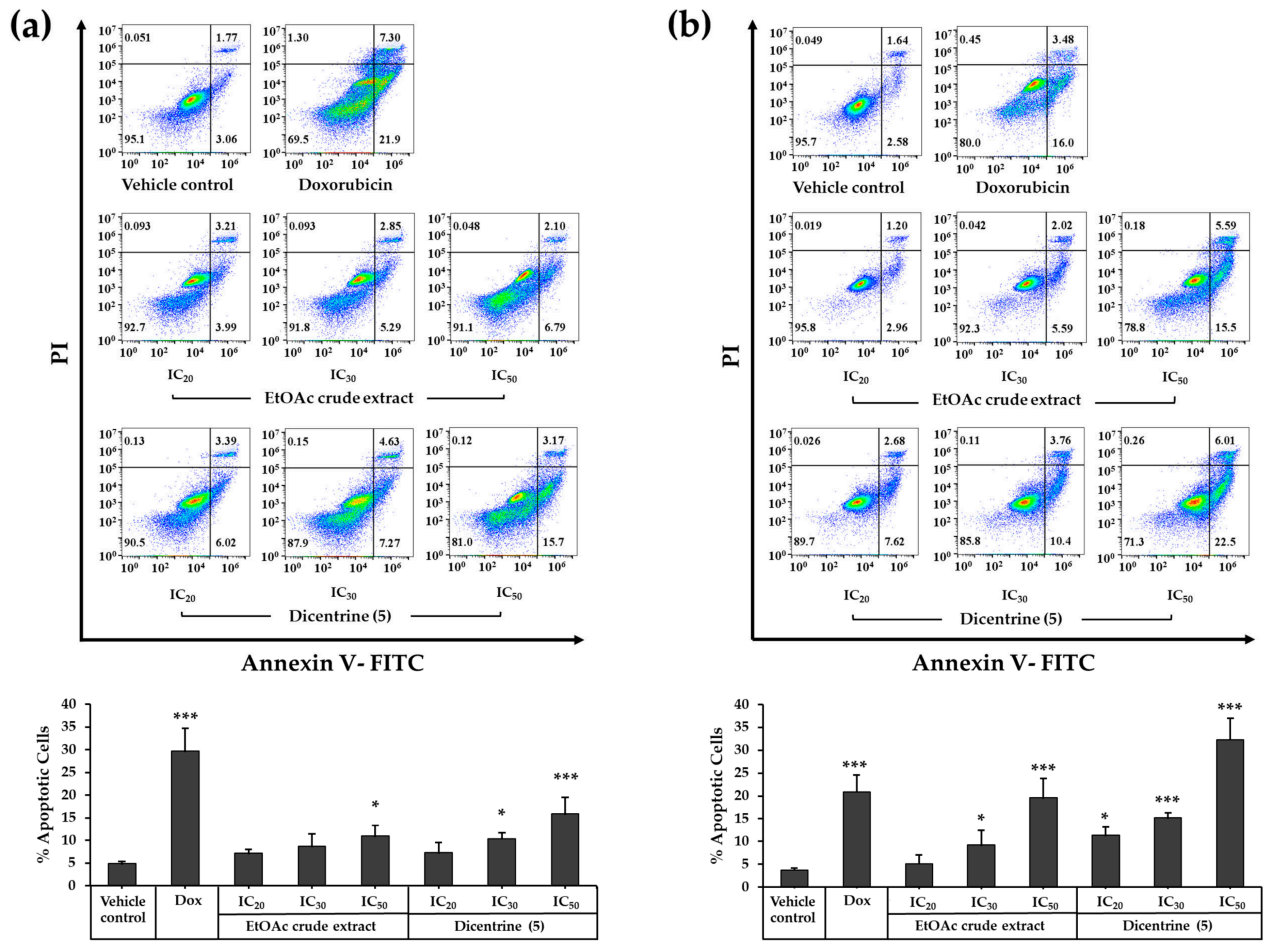
Effects of EtOAc crude extract and dicentrine (**5**) on apoptosis in Raji and Ramos lymphoma cells. Cells were treated with IC_20_, IC_30_, and IC_50_ concentrations for 48 h, and apoptosis was analyzed by Annexin V-FITC/propidium iodide (PI) staining and flow cytometry. (**a**) Raji cells and (**b**) Ramos cells. Doxorubicin served as positive control. Dots represent individual cell events, and the color gradient indicates the relative density of cell populations. Data represent the mean ± SD of three independent experiments. Asterisks indicate statistically significant differences compared with the vehicle control (* *p* < 0.05, *** *p* < 0.001).

Annexin V-FITC/propidium iodide (PI) dual staining, analyzed by flow cytometry was performed to verify the apoptotic response induced by EtOAc crude extract and Dicentrine (**5**). This method distinguishes viable cells (Annexin V-FITC−/PI−) from apoptotic cells, including early apoptotic (Annexin V-FITC+/PI−) and late apoptotic or necrotic cells (Annexin V-FITC+/PI+). For the assay, Raji cells (1.0 × 10^5^ cells/mL) and Ramos cells (1.25 × 10^5^ cells/mL) were treated with EtOAc crude extract and Dicentrine (**5**) at IC_20_, IC_30_, and IC_50_ concentrations for 48 h. Doxorubicin was used as the positive control to validate apoptotic detection. The result demonstrated that both the EtOAc crude extract and dicentrine (**5**) induced apoptosis in a dose-dependent manner in lymphoma cells, with Ramos cells exhibiting greater sensitivity. In Raji cells, significant increases in apoptotic cell populations were observed only at higher concentrations. Treatment with the EtOAc crude extract induced 10.93 ± 2.42% apoptosis at IC_50_ concentration, whereas dicentrine (**5**) significantly increased apoptotic cell populations to 10.36 ± 1.33 and 15.87 ± 3.65% at IC_30_ and IC_50_ concentrations, respectively (Figure 8a). In contrast, Ramos cells exhibited markedly stronger apoptotic responses. Treatment with the EtOAc crude extract resulted in apoptotic populations of 9.21 ± 3.21 and 19.58 ± 4.31% at IC_30_ and IC_50_ concentrations, respectively, while dicentrine (**5**) significantly induced apoptosis to 11.35 ± 1.78, 15.24 ± 1.01, and 32.30 ± 4.66% following treatment at IC_20_, IC_30_, and IC_50_ concentrations for 48 h, respectively (Figure 8b). Notably, the increase in cl-Casp3 expression closely corresponded with the elevation of apoptotic cell populations, supporting its role as a molecular marker of apoptosis induced by these compounds. Collectively, these findings reinforce the biochemical evidence of apoptosis induction and identify dicentrine (**5**) as a more potent inducer of apoptosis in lymphoma cells than the EtOAc crude extract.

### 2.12. Molecular Docking of Dicentrine (***5***) with Lymphoma-Related Targets

**Figure 9:**
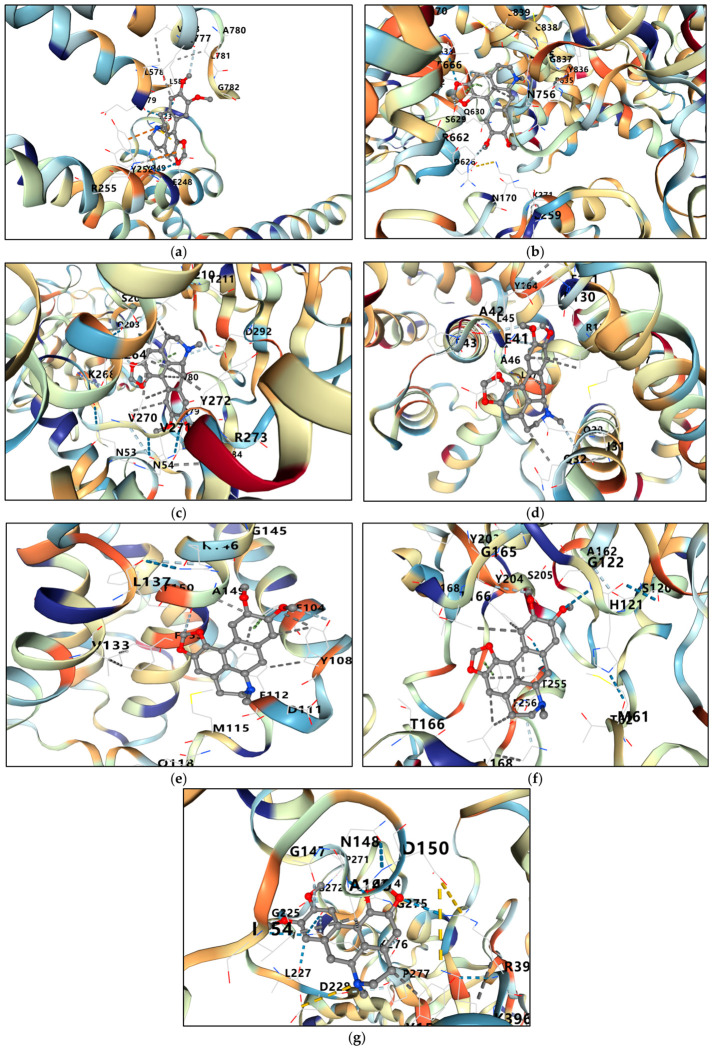
Molecular docking analysis of dicentrine (**5**) with lymphoma-related target proteins. Representative binding poses of dicentrine (**5**) within the binding pockets of (**a**) c-Myc, (**b**) PI3K, (**c**) Akt, (**d**) Bax, (**e**) Bcl2, (**f**) Casp3, and (**g**) Casp9 are shown. Dicentrine (**5**) was stably accommodated within the binding sites of all investigated proteins through multiple non-covalent interactions, including hydrogen bonds, hydrophobic interactions, and van der Waals forces involving key amino acid residues. Among all targets, dicentrine (**5**) exhibited the strongest binding affinity toward Akt, followed by PI3K, Casp9, Casp3, Bax, c-Myc, and Bcl2, supporting its potential involvement in modulation of the PI3K/Akt signaling pathway and mitochondria-mediated apoptosis. Different colors represent different atom types in the protein–ligand complex according to the molecular visualization software and are used only for visualization.

To explore the potential molecular mechanisms underlying the anti-lymphoma activity of dicentrine (**5**), molecular docking analysis was performed against key proteins involved in cell survival and apoptosis, including c-Myc, PI3K, Akt, Bcl2, Bax, caspase-3 (Casp3), and caspase-9 (Casp9). The calculated binding energies are summarized in Table 3. Among the investigated targets, dicentrine (**5**) exhibited the strongest binding affinity toward Akt, with a binding energy of −10.7 kcal/mol, followed by PI3K (−8.9 kcal/mol), Casp9 (−8.5 kcal/mol), Casp3 (−8.2 kcal/mol), Bax (−8.1 kcal/mol), c-Myc (−7.2 kcal/mol), and Bcl2 (−7.0 kcal/mol). The docking conformation of dicentrine (**5**) within the c-Myc binding pocket revealed stable accommodation through multiple non-covalent interactions involving key residues, including Tyr252, Arg255, and Glu248, together with surrounding hydrophobic residues such as Leu578, Leu579, Val777, Ala780, Leu781, and Gly782 (Figure 9a). In PI3K, dicentrine (**5**) was favorably positioned within the kinase binding cleft and interacted with several important amino acid residues, including Asp626, Ser629, Gln630, Arg662, Asn756, Tyr836, Gly837, and Cys838 (Figure 9b), supporting its strong binding affinity. Notably, dicentrine (**5**) demonstrated the highest binding stability toward Akt (Figure 9c), where the ligand was deeply embedded within the active pocket and formed multiple interactions with residues Gln203, Lys268, Val270, Val271, Tyr272, Arg273, Asp292, Asn53, and Asn54. These findings suggest that dicentrine (**5**) may interfere with the PI3K/Akt/c-Myc signaling pathway, which plays a critical role in lymphoma cell survival and proliferation. In addition, dicentrine (**5**) exhibited favorable interactions with apoptosis-related proteins, including the anti-apoptotic protein Bcl2 (Figure 9e) and the pro-apoptotic protein Bax (Figure 9d). Dicentrine (**5**) also showed strong binding affinities toward Casp3 and Casp9 (Figure 9f,g), suggesting its potential involvement in the intrinsic mitochondria-mediated apoptotic pathway. Collectively, these findings indicate that dicentrine (**5**) may exert anti-lymphoma effects through simultaneous modulation of c-Myc signaling, suppression of the PI3K/Akt survival pathway, and activation of apoptosis-associated proteins.

### 2.13. Target Prediction and Screening Using Network Pharmacology

#### 2.13.1. Prediction of Protein Targets of Dicentrine (**5**) Isolated from *S. venosa* Against Lymphoma

**Figure 10:**
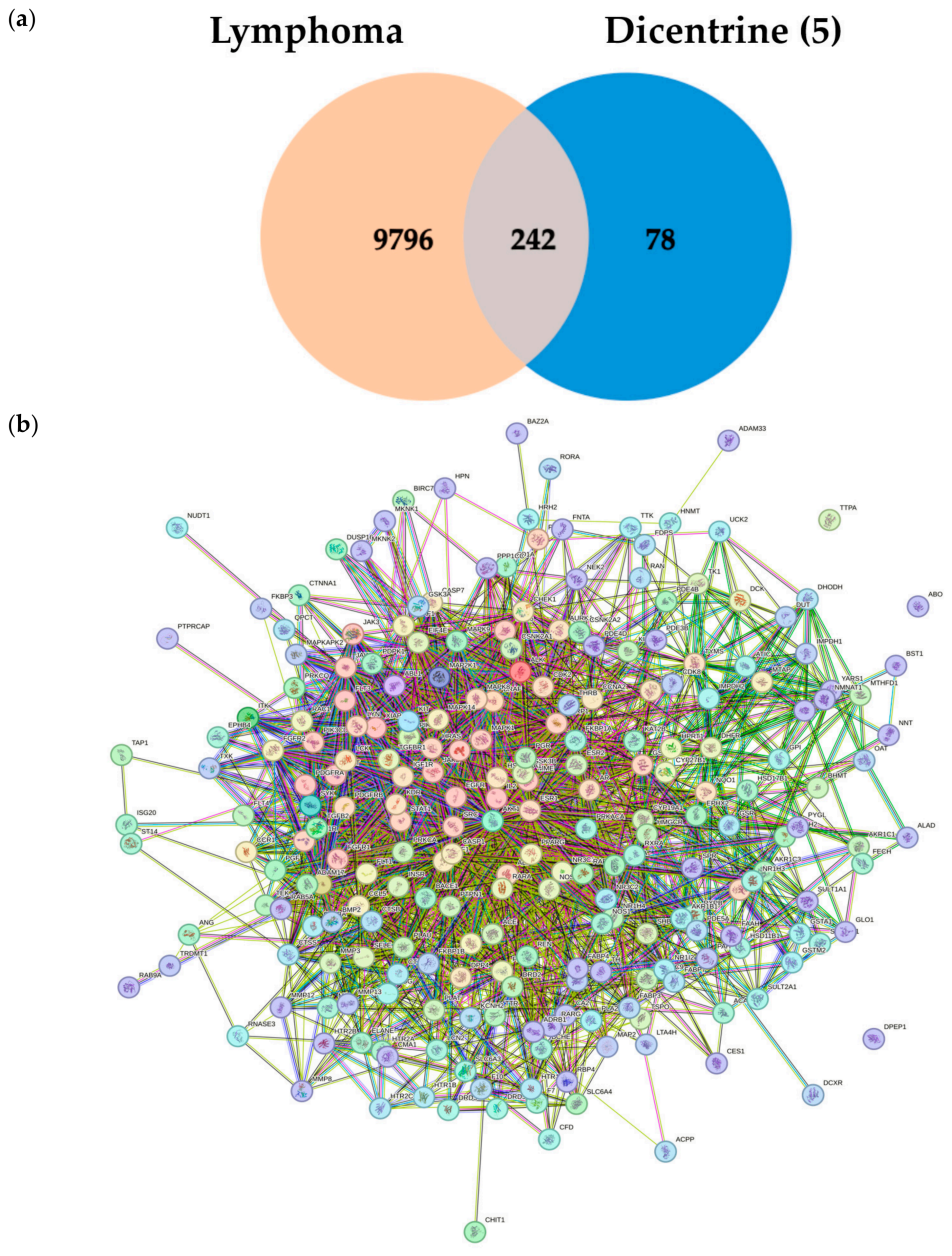

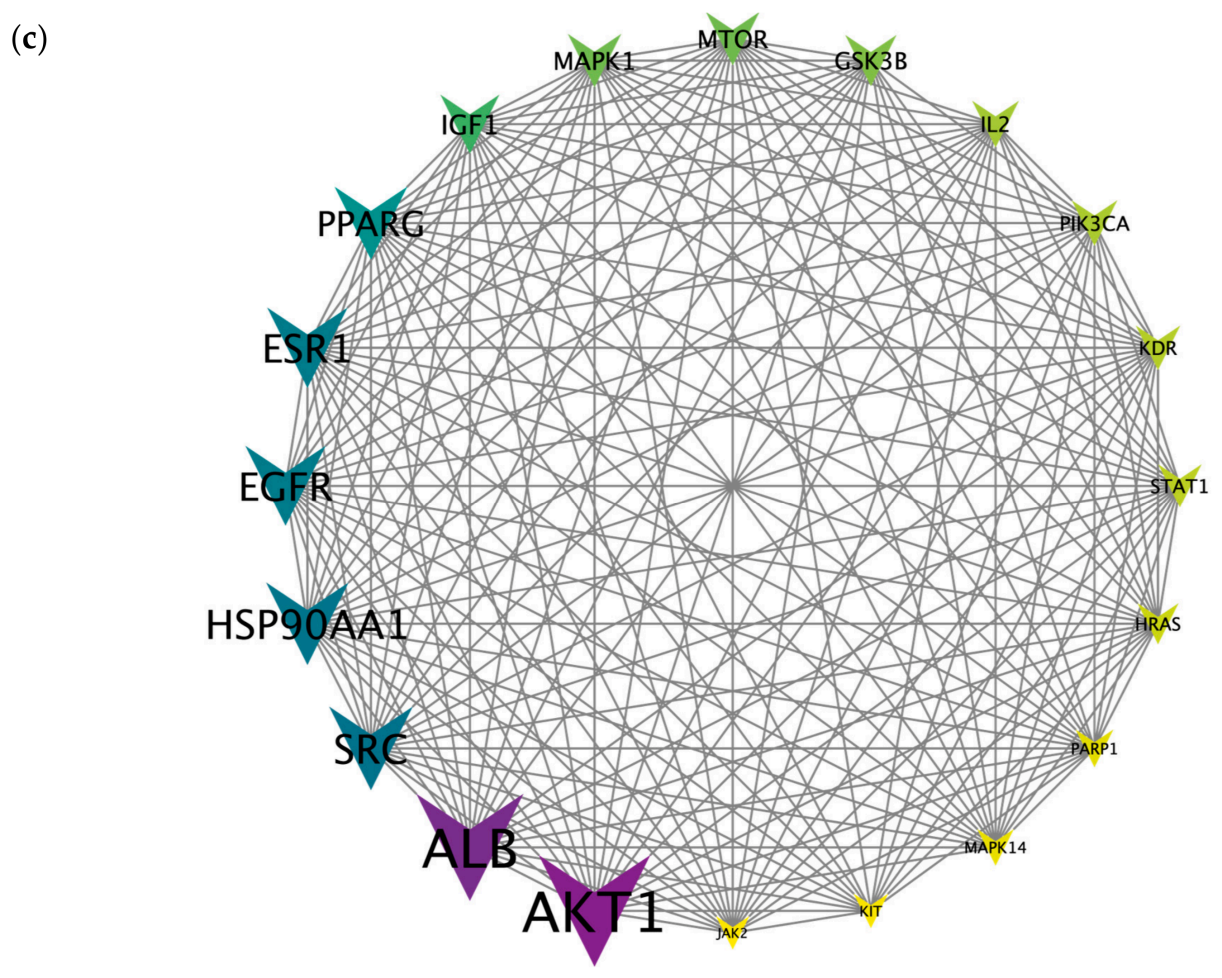
Network pharmacology analysis of the inhibitory effects of dicentrine (**5**) on lymphoma cell growth. (**a**) Venn diagram showing the 242 overlapping target proteins shared between lymphoma-related targets (orange) and dicentrine (**5**)-related targets (blue). (**b**) Protein–protein interaction (PPI) network of the 242 overlapping target proteins. (**c**) Top 20 target proteins nodes with the highest degree values and 180 interaction edges associated with dicentrine (**5**)-mediated inhibition of lymphoma cell growth. Different colors are used only to distinguish the network components for visualization purposes. Larger node labels represent the major hub proteins (targets) associated with dicentrine (**5**).

Based on the potent cytotoxic activity of dicentrine (**5**) isolated from *S. venosa* against lymphoma cell lines, including Raji and Ramos cells, potential protein targets associated with the anti-lymphoma mechanisms of dicentrine (**5**) were predicted using network pharmacology approach. A total of 10,038 lymphoma-related target proteins were collected from the GeneCards and DisGeNet databases. Potential targets of dicentrine (**5**) were predicted using SwissTargetPrediction and PharmMapper databases, yielding a total of 320 target proteins. Among these, 242 overlapping target proteins were identified as common targets between dicentrine (**5**) and lymphoma (Figure 10a). The 242 overlapping target proteins were subsequently analyzed using the STRING database to construct a protein–protein interaction (PPI) network and predict the most relevant proteins associated with both dicentrine (**5**) activity and lymphoma progression (Figure 10b). To identify the most highly interconnected targets, the PPI network was further analyzed and ranked according to degree values. The top 20 target protein nodes with the highest degree values were selected and visualized using Cytoscape version 3.10.4, generating a network containing 180 interaction edges (Figure 10c). Several key target proteins associated with lymphoma cell proliferation and dicentrine (**5**) activity were identified, including Akt1, ALB, SRC, HSP90AA1, EGFR, ESR1, MAPK1, and mTOR. Notably, Akt1 exhibited the highest degree values, suggesting that it may serve as a central regulatory protein involved in dicentrine (**5**)-mediated suppression of lymphoma cell growth.

#### 2.13.2. Gene Ontology (GO) and KEGG Pathway Enrichment Analyses of Overlapping Targets Between Dicentrine (**5**) and Lymphoma

**Figure 11:**
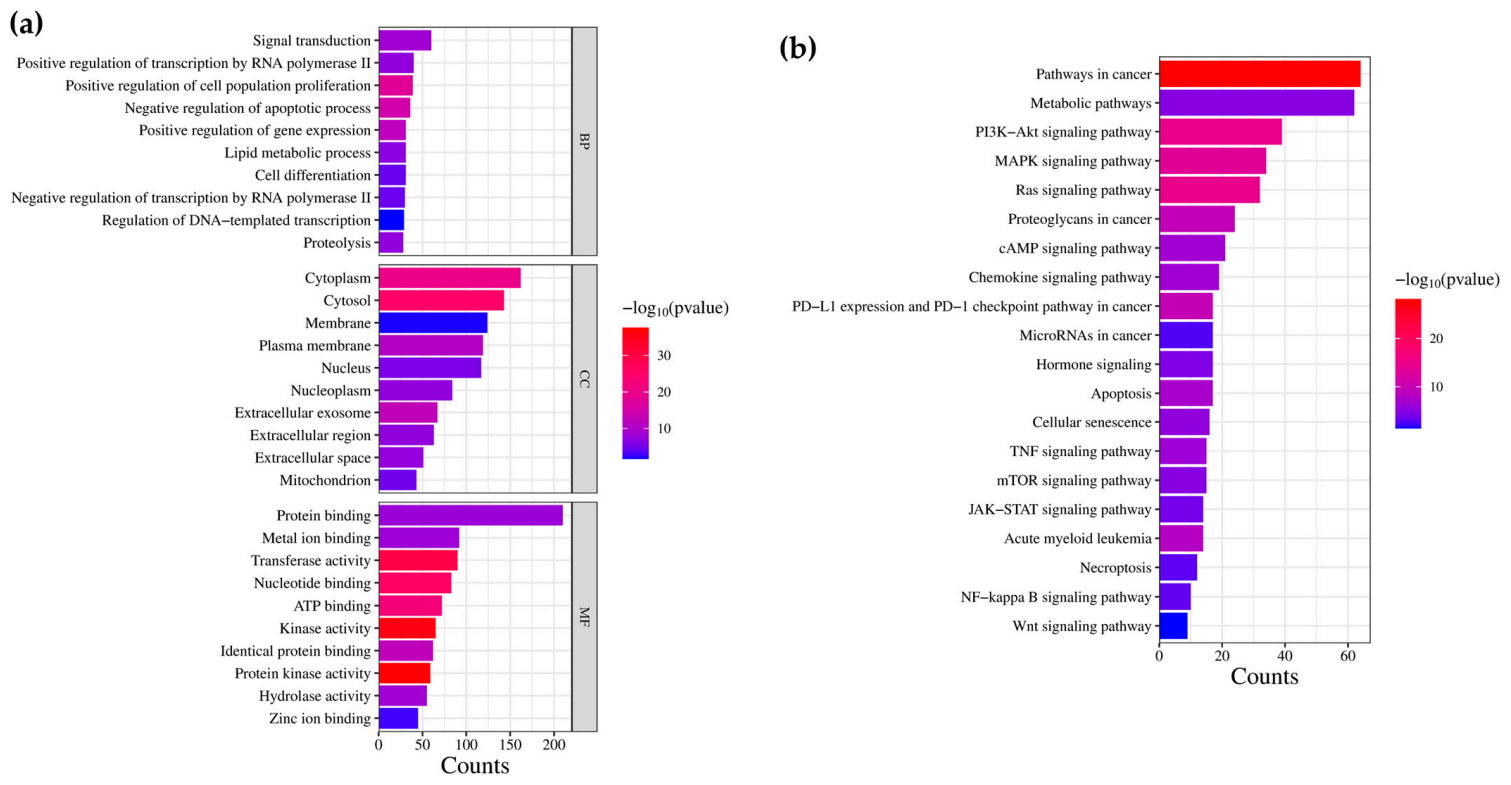
Gene Ontology (GO) and Kyoto Encyclopedia of Genes and Genomes (KEGG) pathway enrichment analyses of the overlapping target proteins associated with the inhibitory effects of dicentrine (**5**) on lymphoma cell proliferation. (**a**) GO enrichment analysis of the overlapping target proteins, include three categories: biological process (BP), cellular component (CC), and molecular function (MF). (**b**) Top 20 enriched KEGG pathways showing statistically significant enrichment at *p* < 0.05.

To further elucidate the biological functions and signaling pathways associated with the 242 overlapping target proteins, Gene Ontology (GO) enrichment and Kyoto Encyclopedia of Genes and Genomes (KEGG) pathway analyses were performed using the DAVID database. As shown in Figure 11a, the overlapping targets were enriched in three major GO categories: biological process (BP), cellular component (CC), and molecular function (MF). The enriched biology processes were mainly associated with signal transduction, positive regulation of transcription by RNA polymerase II, positive regulation of cellular proliferation, regulation of apoptotic process, and positive regulation of gene expression. In terms of molecular functions, the target proteins were primarily involved in protein binding, metal ion binding, transferase activity, nucleotide binding, and ATP binding. Furthermore, KEGG pathway enrichment analysis reveals potential signaling pathways associated with the anti-lymphoma activity of dicentrine (**5**). Among the 178 enriched KEGG pathways, the top 20 high-count pathways included pathways in cancer, metabolic pathways, PI3K-Akt signaling pathway, MAPK signaling pathway, Ras signaling pathway, proteoglycans in cancer, and apoptosis, all of which were significantly enriched (*p* < 0.05) (Figure 11b). These findings suggest that AKT1 and the PI3K-Akt signaling pathway may play central roles in the suppression of lymphoma cell proliferation mediated by dicentrine (**5**).

## 3. Discussion

Lymphoma remains one of the major hematological malignancies worldwide, and despite substantial advances in chemotherapy, targeted therapy, and immunotherapy, treatment resistance, relapse, and systemic toxicity continue to pose major clinical challenges [24,25]. Consequently, the identification of novel anticancer compounds derived from medicinal plants has attracted considerable interest [26,27]. In the present study, dicentrine (**5**) isolated from *Stephania venosa* exhibited potent anti-lymphoma activity through suppression of cell proliferation and induction of apoptosis in Raji and Ramos lymphoma cells. The stronger anti-lymphoma activity observed for the EtOAc extract than for the methanolic extract may be attributed to its enrichment in relatively lipophilic aporphine alkaloids, including dicentrine (**5**). In contrast, the methanolic extract likely contains a broader range of polar constituents that may reduce the relative abundance of these bioactive alkaloids. Among the isolated alkaloids, dicentrine (**5**) demonstrated the strongest cytotoxic activity together with favorable selectivity indices toward lymphoma cells relative to normal PBMCs, highlighting its selective anticancer potential. The present findings are consistent with previous studies demonstrating the anticancer properties of dicentrine (**5**) in several solid tumor models [9,12]. Earlier reports showed that dicentrine (**5**) suppresses cancer cell survival and invasion through modulation of apoptosis-related proteins and inhibition of multiple survival signaling pathways, including Akt, MAPK, NF-κB, and AP-1 signaling [12]. In the current study, dicentrine (**5**) markedly reduced the expression of c-Myc and p-c-Myc in lymphoma cells. c-Myc is a critical oncogenic transcription factor that regulates multiple cellular processes, including cell cycle progression, proliferation, metabolism, and apoptosis [23]. Dysregulated c-Myc expression is strongly associated with aggressive lymphoma progression and poor clinical outcomes, particularly in Burkitt’s lymphoma characterized by c-Myc translocation [28,29]. Therefore, suppression of c-Myc signaling represents an important therapeutic strategy in lymphoma treatment.

Consistent with the reduction in c-Myc expression, dicentrine (**5**) significantly inhibited lymphoma cell proliferation and reduced the number of viable cells in both Raji and Ramos lymphoma cells. Interestingly, the reduction in viable cell populations was more pronounced than the increase in non-viable cells at certain concentrations, suggesting that the antiproliferative effects of dicentrine (**5**) initially involve growth suppression and dysregulation of cell cycle progression prior to extensive induction of cell death. Cell cycle analysis further demonstrated that dicentrine (**5**) altered cell cycle progression in a cell type-dependent manner. In Raji cells, dicentrine (**5**) predominantly induced G0/G1 phase arrest, whereas Ramos cells exhibited G2/M phase accumulation together with increased sub-G1 populations. These findings suggest that dicentrine (**5**) may regulate distinct cell cycle checkpoint mechanisms depending on the molecular characteristics of lymphoma subtypes. The stronger apoptotic response observed in Ramos cells may indicate greater susceptibility of these cells to apoptosis-associated signaling pathways.

Induction of apoptosis was further supported by increased Annexin V-positive cell populations together with significant upregulation of cleaved Casp3 protein expression following dicentrine (**5**) treatment. Casp3 is a major executioner caspase responsible for proteolytic cleavage of multiple intracellular substrates during apoptosis [30,31]. The marked elevation of cl-Casp3 expression, particularly in Ramos cells, supports activation of caspase-dependent apoptotic pathways. In addition, molecular docking analysis demonstrated strong binding affinities of dicentrine (**5**) toward apoptosis-related proteins, including Bax, Casp3, and Casp9, suggesting possible involvement of the intrinsic mitochondria-mediated apoptotic pathway [30,31,32]. Collectively, these findings indicate that dicentrine (**5**) suppresses lymphoma cell survival through coordinated modulation of proliferation-associated signaling and apoptosis-associated mechanisms.

Molecular docking and network pharmacology analyses further provided mechanistic insights into the anti-lymphoma activity of dicentrine (**5**). Among the investigated targets, dicentrine (**5**) exhibited the strongest binding affinity toward Akt, together with favorable binding affinity toward PI3K. Consistent with the docking analysis, network pharmacology identified Akt1 as one of the major hub proteins among the overlapping targets shared between dicentrine (**5**) and lymphoma. KEGG pathway enrichment analysis further highlighted the PI3K/Akt signaling pathway as one of the significantly enriched signaling pathways associated with dicentrine (**5**) activity. The PI3K/Akt pathway is a critical survival pathway involved in lymphoma progression, promoting proliferation, metabolism, resistance to apoptosis, and therapeutic resistance [33,34,35]. Importantly, Akt signaling is also known to regulate c-Myc stability and activation [34]. Collectively, these findings suggest that modulation of the PI3K/Akt/c-Myc signaling pathway may contribute to the anti-lymphoma activity of dicentrine (**5**). Molecular docking and network pharmacology analyses suggested that Akt is a potential molecular target involved in the anti-lymphoma activity of dicentrine (**5**), while Western blot analysis further demonstrated that dicentrine (**5**) significantly reduced total Akt protein expression in lymphoma cells, providing experimental support for the involvement of Akt in dicentrine (**5**)-mediated lymphoma suppression. Nevertheless, Akt activity is primarily regulated through phosphorylation at Ser473 and Thr308. Since phosphorylated Akt was not examined in the present study, the current findings do not conclusively demonstrate inhibition of Akt activation. Therefore, further experimental validation, including analysis of p-Akt, PI3K expression and activation, and additional downstream signaling molecules, is required to clarify the direct regulatory effects of dicentrine (**5**) on the PI3K/Akt signaling pathway. Despite these limitations, our findings provide novel mechanistic evidence that dicentrine (**5**) suppresses the Akt/c-Myc-associated proliferative program in Burkitt lymphoma, thereby establishing a foundation for future mechanistic and translational investigations.

Interestingly, dicentrine (**5**) demonstrated stronger anti-lymphoma activity than the crude EtOAc extract, indicating that dicentrine (**5**) is likely one of the principal bioactive constituents responsible for the anticancer effects of *S. venosa*. Nevertheless, the crude extract retained substantial biological activity, suggesting that additional phytochemicals constituents within the extract may contribute synergistically or additively to the overall anti-lymphoma effects. Such synergistic interactions among phytochemical are commonly observed in medicinal plant extracts and alkaloid-rich plant species, including members of the genus *Stephania* [26,27].

Although the present study provides substantial evidence supporting the anti-lymphoma activity of dicentrine (**5**), several limitations should be acknowledged. The current findings were primarily obtained from in vitro lymphoma cell models, and the precise upstream molecular mechanisms regulating c-Myc suppression remain to be fully elucidated. Furthermore, additional studies using animal models and clinical lymphoma specimens are required to validate the therapeutic potential, pharmacokinetics, properties, and safety profile of dicentrine (**5**) in vivo. Future investigations focusing on transcriptomic analysis, mitochondrial dysfunction, ROS generation, and signaling pathway inhibition may provide deeper mechanistic insights into dicentrine (**5**)-mediated lymphoma suppression. Future studies should further investigate whether dicentrine (**5**) regulates other forms of regulated cell death, including ferroptosis, pyroptosis, necroptosis, and autophagy, as well as identify additional lymphoma-specific molecular targets using transcriptomic and proteomic approaches. Such studies would provide a more comprehensive understanding of the molecular mechanisms underlying the anti-lymphoma activity of dicentrine (**5**). Despite the involvement of signaling pathways that are commonly associated with the anticancer activities of natural compounds, the present study provides the first comprehensive molecular pharmacological characterization of dicentrine (**5**) in human lymphoma. By integrating experimental validation with molecular docking and network pharmacology analyses, our findings demonstrate that dicentrine (**5**) suppresses the c-Myc-associated proliferative program, inhibits lymphoma cell proliferation, and induces apoptosis in Burkitt lymphoma cells, thereby providing new mechanistic evidence supporting its potential for further development as a natural therapeutic agent for lymphoma.

**Figure 12:**
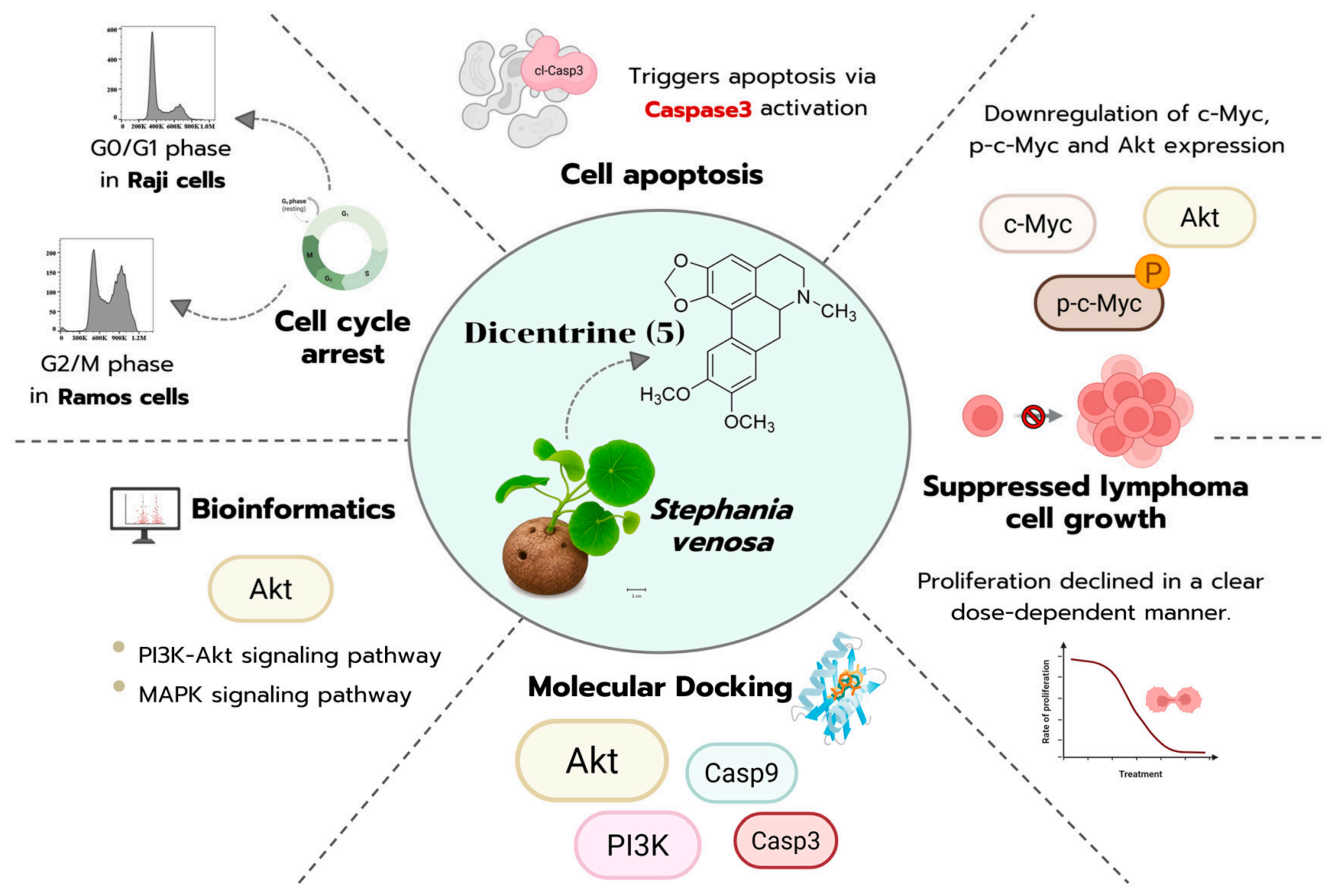
Schematic summary of the proposed anti-lymphoma mechanisms of dicentrine (**5**) isolated from *S. venosa*. Dicentrine (**5**) suppresses lymphoma cell growth through multiple coordinated mechanisms, including inhibition of c-Myc and p-c-Myc, and Akt expression, induction of cell cycle arrest, and activation of Casp3-mediated apoptosis. Dicentrine (**5**) induces G0/G1 phase arrest in Raji cells and G2/M phase arrest in Ramos cells, resulting in dose-dependent suppression of lymphoma cell proliferation. Molecular docking analysis demonstrated favorable interactions of dicentrine (**5**) with Akt, PI3K, Casp3, and Casp9, while network pharmacology analysis identified the PI3K-Akt and MAPK signaling pathways as potential regulatory pathways associated with its anti-lymphoma activity. The proposed mechanism integrates the experimental findings of the present study with molecular docking and bioinformatic analyses. Arrows indicate the proposed regulatory relationships and biological effects of dicentrine (**5**), while different colors are used only to distinguish the illustrated molecular targets and biological processes.

Overall, the present study demonstrates that dicentrine (**5**) isolated from *S. venosa*, possesses potent anti-lymphoma activity through suppression of cell proliferation, induction of cell cycle arrest, and activation of apoptosis. These biological effects are associated with suppression of c-Myc expression and reduced Akt protein expression, while molecular docking and network pharmacology analyses further suggest the potential involvement of the PI3K/Akt pathway. The proposed molecular mechanisms underlying the anti-lymphoma activity of dicentrine (**5**) are schematically summarized in Figure 12. Collectively, these findings highlight dicentrine (**5**) as a promising natural bioactive compound and provide mechanistic insights supporting its further development as a potential therapeutic candidate for lymphoma.

## 4. Materials and Methods

### 4.1. Chemical Materials

3-(4,5-Dimethylthiazol-2-yl)-2,5-diphenyltetrazolium bromide (MTT) was purchased from Sigma-Aldrich (St. Louis, MO, USA). RPMI-1640 medium, Dulbecco’s Phosphate-Buffered Saline (DPBS), Penicillin (10,000 U/mL)/Steptomycin (10,000 μg/mL) and 200 mM L-Glutamine were purchased from GIBCO Invitrogen™ (Grand Island, NY, USA). Fetal bovine serum (FBS) was obtained from Capricorn Scientific (Ebsdorfergrund, Germany). Spectra™ Multicolor Broad Range Protein Ladder was purchased from Thermo Fisher Scientific (Waltham, MA, USA). 30%T Acrylamide/Bis-acrylamide (37.5:1) solution was purchased from PanReact AppliChem (Darmstadt, Germany). 1 M Tris-HCl, pH 8 solution, and 0.5 M Tris-HCl, pH 6.8 solution were purchased from Bio-Rad Laboratories (Richmond, CA, USA). Ethanol (EtOH), methanol (MeOH), ethyl acetate (EtOAc), *n*-hexane (*n*-Hex), and dimethyl sulfoxide (DMSO) were purchased from Labscan (Dublin, Ireland). Silica gel 60 was purchased from Merck (Darmstadt, Germany).

### 4.2. Plant Materials

The tubers of *S. venosa* were collected from Sakon Nakhon Province of Thailand. The plant material was taxonomically authenticated by Dr. Narong Nantasean, and voucher specimen (BKF no. 198608) was deposited at the Forest Herbarium, Department of National Park, Wildlife and Plant Conservation, Ministry of Natural Resources and Environment, Bangkok, Thailand. After collection, the tubers were air-dried, pulverized into a fine powder, and subsequently extracted with ethyl acetate (EtOAc) followed by methanol (MeOH) according to solvent polarity. The obtained extracts were filtered, concentrated under reduced pressure using a rotary evaporator, and dried to remove residual solvents. The crude extracts were subsequently used for biological evaluation of their antiproliferative activity against humam lymphoma cells.

### 4.3. Extraction and Isolation Procedure

**General procedures:** Chromatography: Column chromatography (CC) was performed on silica gel Merck 60 (0.040–0.063 mm). All thin-layer chromatography analyses were carried out on TLC Silica gel 60 F_254_ (Merck) and detected with UV at 254 and 365 nm.

**NMR spectroscopy**: The ^1^H-NMR (500 MHz) and ^13^C-NMR (125 MHz) were recorded on a Bruker AV-500 spectrometer at the Faculty of Science, Chiang Mai University. The spectra were run as CDCl_3_ solution and were referenced to CHCl_3_ as an internal standard (^1^H = d 7.26; ^13^C = 77.0 ppm).

Air-dried tubers of *S. venosa* (6.0 kg) were pulverized and sequentially extracted with ethyl acetate (12 L × 3 days × 3 times) followed by methanol (12 L × 3 days × 3 times) at room temperature (30–35 °C), respectively. The powder of *S. venosa* was placed in the white cotton bag and soaked in each solvent in a stainless-steel tank. After 3 days of maceration, the bag was turned over, and the solvent was drained off. The combined extracts were concentrated under reduced pressure at 40 °C to obtain crude EtOAc (148.58 g) and MeOH (61.89 g) extracts, respectively.

A portion of the EtOAc extract (11.98 g) was subjected to silica gel column chromatography and eluted using a stepwise gradient of *n*-hexane-EtOAc. Elution was initiated with 100% *n*-hexane (500 mL), followed by *n*-hexane-ethyl acetate mixtures of 95:5 (500 mL), 90:10 (1000 mL), 85:15 (500 mL), 80:20 (500 mL), 70:30 (500 mL), 60:40 (300 mL), 50:50 (300 mL), 40:60 (500 mL), 20:80 (300 mL), and 0:100 (300mL). Subsequently, the polarity of the mobile phase was increased using ethyl acetate-methanol mixtures of 95:5 (200 mL), and 90:10 (200 mL, *v*/*v*). Eluates were collected sequentially and combined based on their TLC profiles to afford ten fractions (Fractions A1–10). Compound **2** (365.0 mg, 0.0755% *w*/*w*) was obtained from fraction A5.

Fraction A2 (0.3247 g) was further purified by silica gel column chromatography and eluted using a stepwise gradient of *n*-hexane-dichloromethane, followed by dichloromethane-methanol. Elution was initiated with *n*-hexane-dichloromethane (95:5, 150 mL), followed by 90:10 (300 mL), 85:15 (300 mL), 80:20 (100 mL), 70:30 (100 mL), 60:40 (100 mL), 50:50 (100 mL), 30:70 (100 mL), and 0:100 (200 mL, *v*/*v*). Subsequently, the polarity of the mobile phase was increased using dichloromethane-methanol mixtures of 98:2 (100 mL), 95:5 (100 mL), and 90:10 (200 mL, *v*/*v*). Eluates were collected sequentially and combined based on a TLC pattern and concentrated to give two fractions (B2.1–B2.2). Compound **1** (12.2 mg, 0.0025% *w*/*w*) was obtained from subfraction B2.1.

Fraction A6 (1.26 g) was recrystallized from EtOH (10 mL). The mixture was heated until the solid dissolved completely and was then allowed to cool slowly to room temperature. Upon standing, a yellow precipitate formed. The precipitate was collected to afford Compound **3** (471.6 mg, 0.0976% *w*/*w*) as a yellow solid.

Fraction A8 (0.48 g) was further purified by silica gel column chromatography. Elution was performed with a hexane-acetone gradient system of increasing polarity. The column was successively eluted with hexane/acetone mixtures of 80:20, 70:30, and 60:40 (*v*/*v*), using 200 mL of each solvent system. The collected fractions were combined based on a TLC pattern and concentrated to give four fractions (C8.1–C8.4). Compound **4** (256.9 mg, 0.0531% *w*/*w*) was obtained from subfraction C8.2.

Fraction A10 (2.87 g) was recrystallized from EtOH (20 mL). The mixture was heated until the solid dissolved completely and was then allowed to cool slowly to room temperature. Upon standing, a brown precipitate formed. The solid was collected to afford Compound **5** (756.8 mg, 0.1565% *w*/*w*) as a brown solid.

The purity of the isolated compounds was evaluated by TLC, ^1^H and ^13^C NMR spectroscopy, indicating apparent purity, as no significant impurity signals were observed. The corresponding ^1^H and ^13^C NMR spectra are shown in Figures S1–S10. Subsequently, the purified compounds were analyzed by GC-MS, and the corresponding chromatograms are shown in Figures S11–S15.

### 4.4. Cells and Cell Culture Conditions

Human Burkitt lymphoma-derived Raji cells (EP-CL-0189; Elabscience, Houston, TX, USA) and Ramos cells (iCell-h179; iCell Bioscience, Shanghai, China) cell lines were used in this study. Cells were maintained in RPMI-1640 medium (Roswell Park Memorial Institute, Invitrogen™, Carsbad, CA, USA) supplemented with 10% fetal bovine serum (FBS), 2 mM L-glutamine, 100 units/mL penicillin, and 100 μg/mL streptomycin (Invitrogen™, Carsbad, CA, USA) at 37 °C in a humidified incubator containing 5% CO_2_.

### 4.5. Isolation of Peripheral Blood Mononuclear Cells (PBMCs)

Peripheral blood mononuclear cells (PBMCs) were isolated from whole blood collected from at least five healthy volunteers using Ficoll-Hypaque density gradient centrifugation. The study involving human participants was approved by the Human Research Ethics Committee of the Faculty of Associated Medical Sciences, Chiang Mai University (Approval No. 246/2026; approved on 29 May 2026). Written informed consent was obtained from all volunteers before blood collection, and all experimental procedures were conducted in accordance with the approved ethical guidelines and relevant regulations. For PBMC isolation, whole blood samples (10–20 mL) were diluted 1:1 with sterile phosphate-buffered saline (PBS; pH 7.4) and carefully layered onto Ficoll-Hypaque solution. Following centrifugation at 400× *g* for 30 min at room temperature, the mononuclear cell layer was carefully collected and washed twice with PBS by centrifugation at 2000 rpm for 10 min. Cell number and viability were subsequently determined using the trypan blue exclusion assay.

### 4.6. Cytotoxicity Analysis Using MTT Assay

The cytotoxic activities of *S. venosa* crude extracts and purified compounds against lymphoma cells were determined using the MTT (3-(4,5-dimethylthiazol-2-yl)-2,5-diphenyltetrazolium bromide) colorimetric assay. Raji and Ramos cells were seeded at densities of 1.0 × 10^5^ and 1.25 × 10^5^ cells/mL, respectively, and treated with various concentrations (0–100 µg/mL) of crude extract or isolated compounds for 48 h. Cell viability was then evaluated according to the MTT assay described previously [36]. Following treatment, 15 µL of MTT solution (5 mg/mL) was added to each well, and the plates were incubated for an additional 4 h at 37 °C to allow intracellular reduction of MTT into insoluble formazan crystals. The culture medium was then replaced with dimethyl sulfoxide (DMSO) to dissolve the formazan crystals, and the plates were gently agitated until complete solubilization was achieved. Absorbance was recorded at 578 nm with a reference wavelength of 630 nm using a Beckman Coulter DTX880 microplate reader (Fullerton, CA, USA). Cell viability was calculated according to Equation (1), with vehicle-treated cells (DMSO) defined as 100% viability:

(1) $$\%\;\mathrm{Cell}\;\mathrm{viability}=\;\frac{\mathrm{Mean}\;\mathrm{absorbance}\;\mathrm{in}\;\mathrm{test}\;\mathrm{well}}{\mathrm{Mean}\;\mathrm{absorbance}\;\mathrm{in}\;\mathrm{vehicle}\;\mathrm{control}\;\mathrm{well}}\;\times \;100$$

Dose–response curves were generated from three independent experiments, and the half-maximal inhibitory concentration (IC_50_) values were calculated and expressed as mean ± standard deviation (SD).

### 4.7. Selectivity Index

To evaluate the preferential cytotoxicity of the crude extracts and isolated compounds toward lymphoma cells relative to normal cells, the selectivity index (SI) was determined. The SI was calculated as the ratio of half-maximal inhibitory concentration (IC_50_) in PBMCs to that obtained in lymphoma cells (Raji or Ramos), as shown in Equation (2) [37,38]. An SI value greater than 1 indicates that a compound exhibits greater cytotoxicity toward cancer cells than toward normal cells.

(2) $$\mathrm{Selectivity}\;\mathrm{index}\;=\frac{{\mathrm{IC}}_{50\;\mathrm{PBMC}}}{{\mathrm{IC}}_{50\;\mathrm{Lymphoma}}}$$

where IC_50 PBMC_ represents the IC_50_ value determined in PBMCs, whereas IC_50 Lymphoma_ represents the IC_50_ value obtained in Raji and Ramos cells under the same experimental conditions.

### 4.8. Trypan Blue Exclusion Assay

Cell proliferation and viability were evaluated using the trypan blue exclusion method after treatment with the indicated compounds. Following treatment, cell suspensions were mixed with 0.2% trypan blue solution, and viable and non-viable cells were distinguished based on dye exclusion. Unstained cells were considered viable, whereas blue stained cells were regarded as non-viable. Cell numbers were manually determined using a hemocytometer under a light microscope. The percentages of viable cells and cell proliferation were subsequently calculated. All experiments were performed independently in triplicate.

### 4.9. Network Pharmacology and Bioinformatic Analysis

To investigate the molecular mechanisms underlying the anti-lymphoma activity of dicentrine (**5**), an integrated workflow combining network pharmacology and molecular docking analyses was performed. Candidate protein targets of dicentrine (**5**) were predicted using the PharmMapper and SwissTargetPrediction databases, whereas lymphoma-associated target genes were retrieved from the GeneCards and DisGeNET databases. The intersecting targets shared between dicentrine (**5**) and lymphoma were identified and subjected to downstream function analyses. Gene Ontology (GO) annotation and Kyoto Encyclopedia of Genes and Genomes (KEGG) pathway enrichment analyses were conducted using the DAVID Bioinformatics Resources platform (https://davidbioinformatics.nih.gov/ (accessed on 6 February 2026)). GO enrichment classified the overlapping targets into Biological Process (BP), Cellular Component (CC), and Molecular Function (MF), Whereas KEGG analysis was used to identify signaling pathways potentially involved in lymphoma progression, cell proliferation, and apoptosis. The top 20 proteins with the highest degree centrality values in protein–protein interaction (PPI) network were selected as hub targets for further investigation. GO and KEGG enrichment results were visualized using the SRplot platform (https://www.bioinformatics.com.cn/srplot (accessed on 6 February 2026)), and statistical significance was expressed as −log10 (*p*-value).

The PPI network was generated using STRING version 12.0 (https://string-db.org/ (accessed on 1 April 2026)) and visualized with Cytoscape version 3.10.3. Molecular docking analysis was subsequently performed using the CB-Dock2 server to evaluate the binding interactions between dicentrine (**5**) and selected lymphoma-related proteins. The three-dimensional structure of dicentrine (**5**) was obtained from the PubChem database (https://pubchem.ncbi.nlm.nih.gov/ (accessed on 1 April 2026)), whereas protein crystal structures were retrieved from the Protein Data Bank (PDB), including c-Myc (PDB ID: 1NKP), AKT1 (PDB ID: 8UW7), PIK3CA (PDB ID: 8EXL), BCL2 (PDB ID: 6GL8), BAX (PDB ID: 4S0O), Casp3 (PDB ID: 3DEI), and Casp9 (PDB ID: 1JXQ). Binding cavities were automatically predicted using the built-in cavity detection algorithm implemented in CB-Dock2. Molecular docking was performed using AutoDock Vina version 1.2.0. Binding affinity was evaluated as the AutoDock Vina scores (kcal/moL), and the docking pose with the lowest binding energies for each protein was selected for visualization using PyMOL (version 3.0.4).

### 4.10. Cell Cycle Distribution

Cell cycle analysis was performed by flow cytometry following treatment with the test samples. Raji and Ramos cells were seeded at densities of 1.0 × 10^5^ and 1.25 × 10^5^ cells/mL, respectively, in RPMI-1640 medium containing 0.5% FBS and incubated overnight under serum-restricted conditions before treatment. Cells were subsequently treated with the *S. venosa* EtOAc crude extract or dicentrine (**5**) at concentration corresponding to IC_10_, IC_20_, and IC_30_ for 48 h. Following treatment, cells were harvested, washed twice with cold PBS, pH 7.4, and fixed in 70% ice-cold ethanol for 2 h. After fixation, the cells were washed again with cold PBS, pH 7.4 and stained with 1 × propidium iodide (PI)/RNase staining solution (Abcam, Cambridge, UK) following the manufacturer’s protocol. DNA content was analyzed using a BD Accuti^TM^ C6 plus flow cytometer (BD Biosciences, San Jose, CA, USA). All experiments were performed independently in triplicate.

### 4.11. Apoptosis Analysis

Apoptotic cell death was quantified using the FITC Anexin V Apoptosis Detection Kit with PI (BioLegend, San Diego, CA, USA). Raji and Ramos cells were seeded at densities of 1.0 × 10^5^ and 1.25 × 10^5^ cells/mL, respectively, and treated with the *S. venosa* EtOAc crude extract or dicentrine (**5**) at concentrations corresponding to IC_20_, IC_30_, and IC_50_ for 48 h. Following treatment, cells were collected and stained according to the manufacturer’s protocol. Briefly, cell pellets were resuspended in 100 µL of Annexin V Binding Buffer, Followed by the addition of 5 µL of Annexin V-FITC and 10 µL of PI solution. The samples were gently mixed and incubated for 15 min at room temperature (25 °C) in the dark. After incubation, 400 µL of Annexin V Binding Buffer was added prior to flow cytometric analysis. Apoptotic cell populations were quantified using a BD Accuti^TM^ C6 plus flow cytometer (BD Biosciences, San Jose, CA, USA). All experiments were performed independently in triplicate.

### 4.12. Western Blot Analysis

Western blotting was performed to examine the expression of proteins associated with dicentrine (**5**)-induced apoptosis and cell proliferation. Raji and Ramos cells were treated with the *S. venosa* EtOAc crude extract or dicentrine (**5**) at concentration corresponding to IC_20_, IC_30_, and IC_50_ for 48 h. Following treatment, total cellular proteins were extracted using radioimmunoprecipitation assay (RIPA) buffer supplemented with a protease inhibitor cocktail. Protein concentrations were determined using the Pierce™ BCA Protein Assay Kit (Thermo Fisher Scientific, Waltham, MA, USA). Equal amounts of protein (20 µg) were resolved on 12% sodium dodecyl sulfate-polyacrylamide gel electrophoresis (SDS-PAGE) and electrotransferred onto polyvinylidene difluoride (PVDF) membranes. The membranes were blocked with 5% (*w*/*v*) skim milk prepared in Tris-buffered saline containing 0.1% Tween-20 (TBST) for at least 30 min at room temperature before incubation with the primary antibodies. The following rabbit antibodies were used: anti-c-Myc IgG (1:1000), phospho-c-Myc (Ser62) IgG (1:1000), anti-Akt (pan, C67E7) IgG (1:1000), anti-cl-Casp3 (Asp175, clone 5A1E) IgG (1:1000) (Cell Signaling Technology, Danvers, MA, USA), and anti-GAPDH IgG (1:16,000; MilliporeSigma, Burlington, MA, USA) as the loading control. Membranes were incubated overnight at 4 °C with anti-c-Myc, anti-p-c-Myc, anti-Akt, and anti-cl-Casp3 antibodies, whereas anti-GAPDH was incubated for 1 h at room temperature (25 °C). After washing with TBST, the membranes were incubated with horseradish peroxidase (HRP)-conjugated anti-rabbit IgG secondary antibody (1:20,000; Promega, Madison, WI, USA). Immunoreactive protein bands were detected using Luminata™ Forte Western HRP substrate (Merck, Darmstadt, Germany) and visualized with a ChemiDoc MP Imaging System (Bio-Rad, Hercules, CA, USA). Band intensities were quantified using Image Lab Software version 6.1 (Bio-Rad, Hercules, CA, USA) and normalized to GAPDH. All experiments were performed independently in triplicate.

### 4.13. Statistical Analysis

All experiments were obtained from three independent experiments and are expressed as the mean ± standard deviation (SD). Statistical analyses were performed using IBM SPSS Statistics software version 22.0 (IBM Corp., Armonk, NY, USA). Differences among multiple groups were evaluated by one-way analysis of variance (ANOVA), followed by Bonferroni’s post hoc test for multiple comparisons when appropriate. Statistical significance is indicated as * *p* < 0.05, ** *p* < 0.01, and *** *p* < 0.001.

## 5. Conclusions

In conclusion, dicentrine (**5**) isolated from *S. venosa* exhibited potent anti-lymphoma activity against Raji and Ramos lymphoma cells. Among the isolated alkaloids, dicentrine (**5**) demonstrated the strongest cytotoxic activity together with favorable selectivity toward lymphoma cells relative to normal PBMCs. Dicentrine (**5**) effectively suppressed lymphoma cell proliferation, reduced viable cell numbers, induced cell cycle arrest, and promoted apoptosis in a dose-dependent manner. Mechanistically, dicentrine (**5**) significantly downregulated c-Myc, p-c-Myc, and total Akt protein expression, accompanied by increased cl-Casp3 expression and elevated apoptotic cell populations. Together with molecular docking and network pharmacology analyses, these findings suggest the potential involvement of the PI3K/Akt/c-Myc signaling pathway in the anti-lymphoma activity of dicentrine (**5**). Collectively, these findings provide mechanistic insights into the anti-lymphoma activity of dicentrine (**5**) and support its further development as a promising natural therapeutic candidate for lymphoma.

## Figures and Tables

**Table 1:** IC_50_ values and selectivity indices of two crude extracts from *S. venosa* against lymphoma cell lines and normal PBMCs.

Crude Extraction	IC_50_ Value (µg/mL) (Mean ± SD)			Selectivity Index (SI)	
	Raji	Ramos	PBMCs	Raji	Ramos
MeOH crude extract	47.31 ± 6.02	20.57 ± 0.54	>100	>2.11	>4.86
EtOAc crude extract	24.76 ± 1.70	14.89 ± 1.36	65.91 ± 7.89	2.66	4.42

**Table 2:** IC_50_ values and selectivity indices (SI) of five isolated compounds from the EtOAc crude extract of *S. venosa* against lymphoma cell lines and normal PBMCs.

Compounds	IC_50_ Value (µg/mL) (Mean ± SD)			Selectivity Index (SI)	
	Raji	Ramos	PBMCs	Raji	Ramos
Dehydroisolaureline (**1**)	39.75 ± 7.96	9.56 ± 1.90	84.79 ± 1.10	2.13	8.87
Dehydrodicentrine (**2**)	44.94 ± 3.20	20.16 ± 2.22	>100	>2.23	>4.96
Tetrahydropalmatine (**3**)	88.92 ± 5.14	57.05 ± 2.10	>100	>1.12	>1.75
Crebanine (**4**)	40.94 ± 5.02	23.97 ± 0.63	43.54 ± 3.51	1.06	1.82
Dicentrine (**5**)	9.03 ± 0.53	5.16 ± 0.44	41.44 ± 5.33	4.59	8.03

**Table 3:** Component-target molecular docking of dicentrine (**5**) against c-Myc, PI3K, Akt, Bcl2, Bax, Casp3, and Casp9.

Compound	Binding Energy (kcal/moL)						
	c-Myc	PI3K	Akt	Bcl2	Bax	Casp3	Casp9
Dicentrine (**5**)	−7.2	−8.9	−10.7	−7.0	−8.1	−8.2	−8.5

## Data Availability

The original contributions presented in the study are included in the article/Supplementary Materials, further inquiries can be directed to the corresponding author.

## Supplementary Materials

The following supporting information can be downloaded at https://www.mdpi.com/article/10.3390/ijms27156974/s1.

## Abbreviations

The following abbreviations are used in this manuscript:

Casp3	Caspase-3
Casp9	Caspase-9
cl-Casp3	Cleaved caspase-3
c-Myc	Cellular myelocytomatosis
p-c-Myc	Phosphorylated c-Myc
DLBCL	Diffuse large B-cell lymphoma
GAPDH	Glyceraldehyde 3-phosphate dehydrogenase
GO	Gene Oncology
IC	Inhibitory concentration
KEGG	Kyoto Encyclopedia of Gene and Genome
mTOR	Mammalian target of rapamycin
MTT	3-(4,5-dimethythiazol-2-thizolyl)-2,5-diphenyl tetrazolium bromide
NHL	Non-Hodgkin lymphoma
PPI	Protein–protein interaction
PBMCs	Peripheral blood mononuclear cells
PI3K	Phosphoinositide 3-kinase
SI	Selective index
