# Supplementary material for: Molecular Pharmacological Characterization of Dicentrine Isolated from Stephania venosa in Human Lymphoma Cells
Source: Int J Mol Sci. 2026 Aug 3;27(15):6974. doi: 10.3390/ijms27156974 (PMC13466670; doi:10.3390/ijms27156974)
Supplement: Supplementary file 1 [file ijms-27-06974-s001.zip › ijms-4438548-supplementary.pdf]

Supplementary data

Molecular Pharmacological Characterization of Dicentrine Isolated from *Stephania venosa* in Human Lymphoma Cells

Aroonchai Saiai [1], Sirinya Moakmamern [2], Lapamas Rueankum [2], Wenxian Yin [2], Singkome Tima [2,3,5], Siriporn Okonogi [3,4], Sawitree Chiampanichayakul [2,3,5], and Songyot Anuchapreeda [2,3,5,*]

[1] Department of Chemistry, Faculty of Science, Chiang Mai University, Chiang Mai 50200, Thailand; aroonchai.s@cmu.ac.th (A.S.)
[2] Department of Medical Technology, Faculty of Associated Medical Sciences, Chiang Mai University, Chiang Mai 50200, Thailand; Lapamas_ru@cmu.ac.th (L.R.); sirinya_moakm@cmu.ac.th (S.M.); yinwenxian@swmu.edu.cn (W.Y.); sawitree.chiampa@cmu.ac.th (S.C.)
[3] Center of Excellence in Pharmaceutical Nanotechnology, Chiang Mai University, Chiang Mai 50200, Thailand; okng2000@gmail.com (S.O.)
[4] Department of Pharmaceutical Sciences, Faculty of Pharmacy, Chiang Mai University, Chiang Mai 50200, Thailand
[5] Cancer Research Unit of Associated Medical Sciences (AMS CRU), Faculty of Associated Medical Sciences, Chiang Mai University, Chiang Mai 50200, Thailand
* Correspondence: songyot.anuch@cmu.ac.th (S.A.); Tel.: +66-5394-9237

Abstract

Lymphoma remains a major hematological malignancy associated with treatment resistance and systemic toxicity, highlighting the need for novel anticancer agents derived from natural products. In this study, dicentrine (**5**), an aporphine alkaloid isolated from *Stephania venosa*, was investigated for its anti-lymphoma activity in Raji and Ramos cells. Among the isolated compounds, dicentrine (**5**) exhibited the strongest cytotoxic activity, with IC_{50} values of 9.03 ± 0.53 and 5.16 ± 0.44 μg/mL in Raji and Ramos cells, respectively, while demonstrating favorable selectivity toward lymphoma cells relative to peripheral blood mononuclear cells (PBMCs). Dicentrine (**5**) significantly suppressed c-Myc and phosphorylated c-Myc expression, reduced lymphoma cell proliferation, and decreased total viable cell numbers in a dose-dependent manner. Cell cycle analysis revealed G0/G1 arrest in Raji cells and G2/M arrest in Ramos cells. Furthermore, dicentrine (**5**) induced apoptosis, as evidenced by increased Annexin V-positive populations and elevated cleaved caspase-3 expression. Molecular docking analysis demonstrated strong binding affinities of dicentrine (**5**) toward Akt, PI3K, caspase-3, and caspase-9, while network pharmacology identified AKT1 and the PI3K/Akt signaling pathway as potential targets associated with lymphoma suppression. Western blot analysis further demonstrated that dicentrine (**5**) significantly reduces total Akt protein expression. Overall, the present findings indicate that dicentrine (**5**) suppresses lymphoma progression by inhibiting cell proliferation and promoting apoptotic cell death, highlighting its potential as a promising natural therapeutic candidate for lymphoma.

Keywords: dicentrine; *Stephania venosa*; cancer; lymphoma; c-Myc; apoptosis; PI3K/Akt signaling

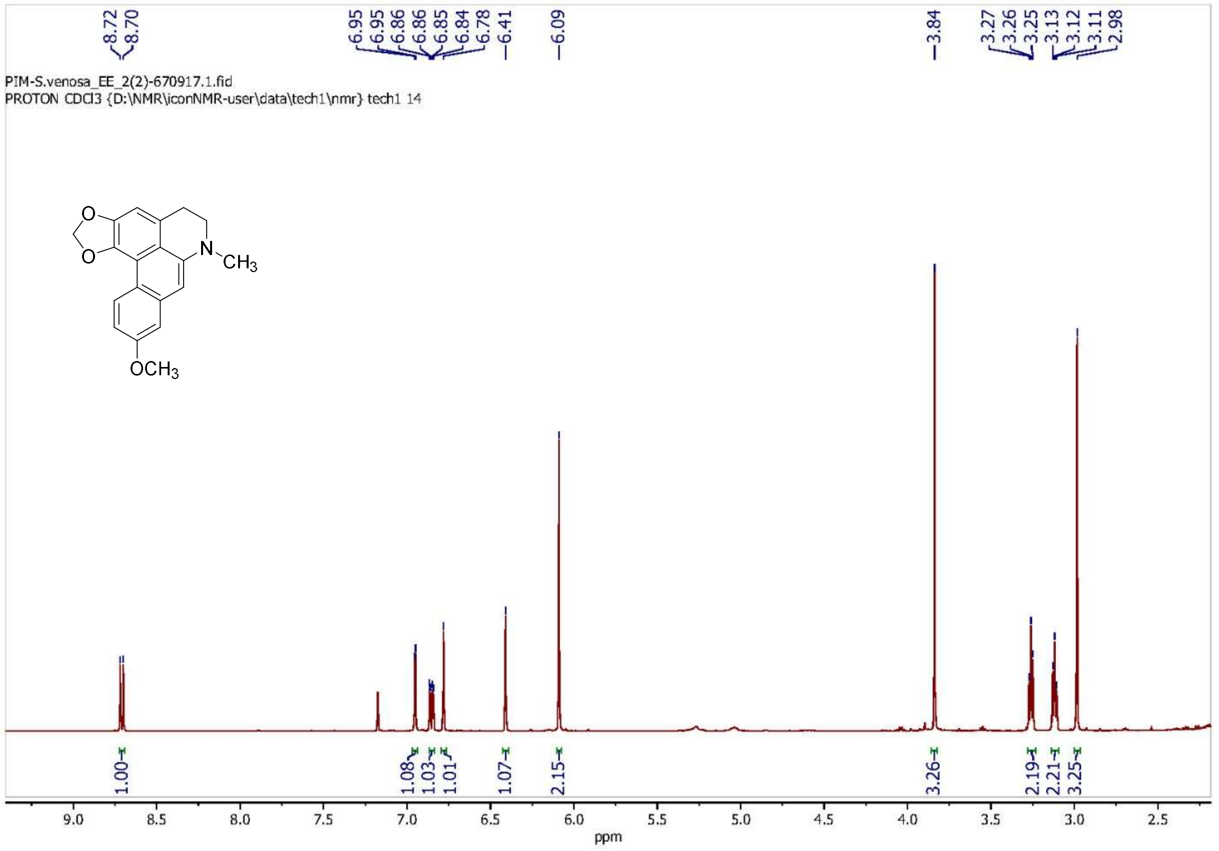

Figure S1. ^{1}H NMR spectrum ($CDCl_3$, 500 MHz) of dehydroisolaureline (**1**)

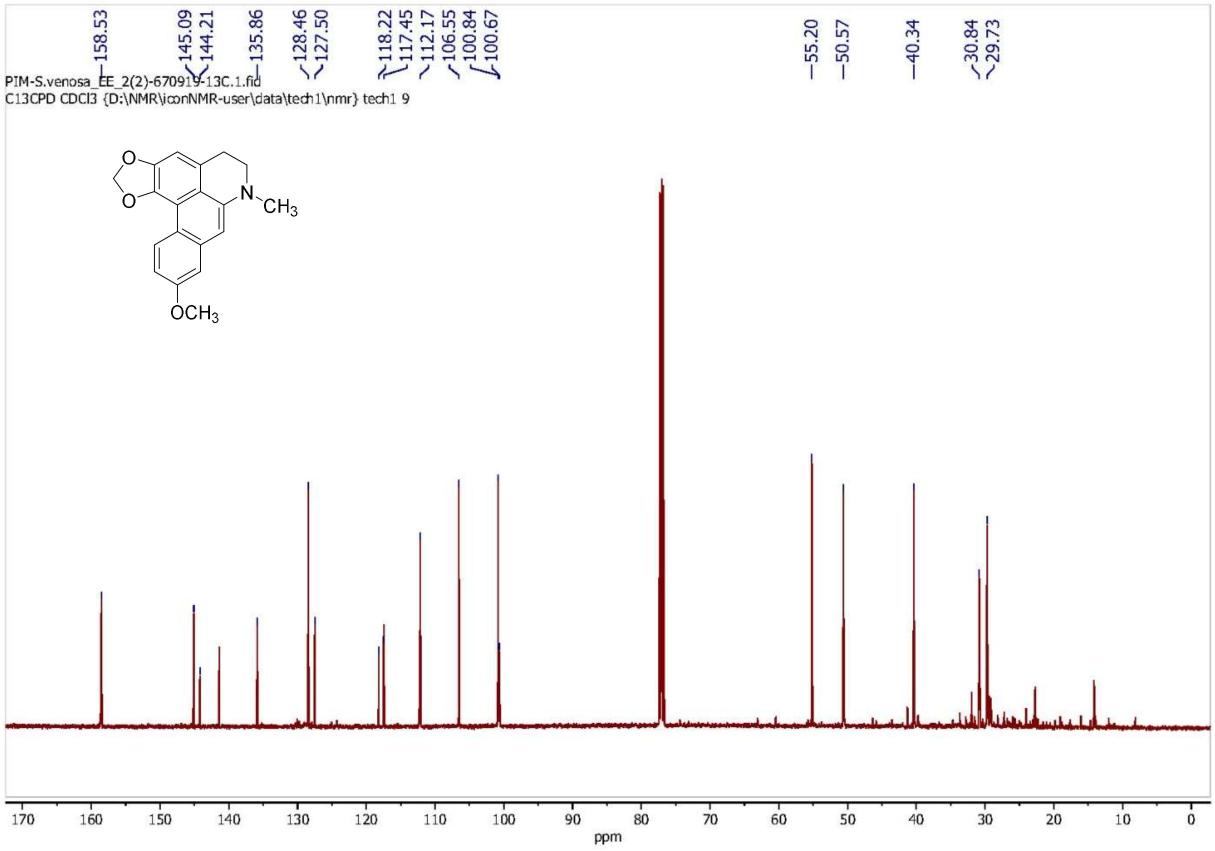

Figure S2. ^{13}C NMR spectrum ($CDCl_3$,125 MHz) of dehydroisolaureline (**1**)

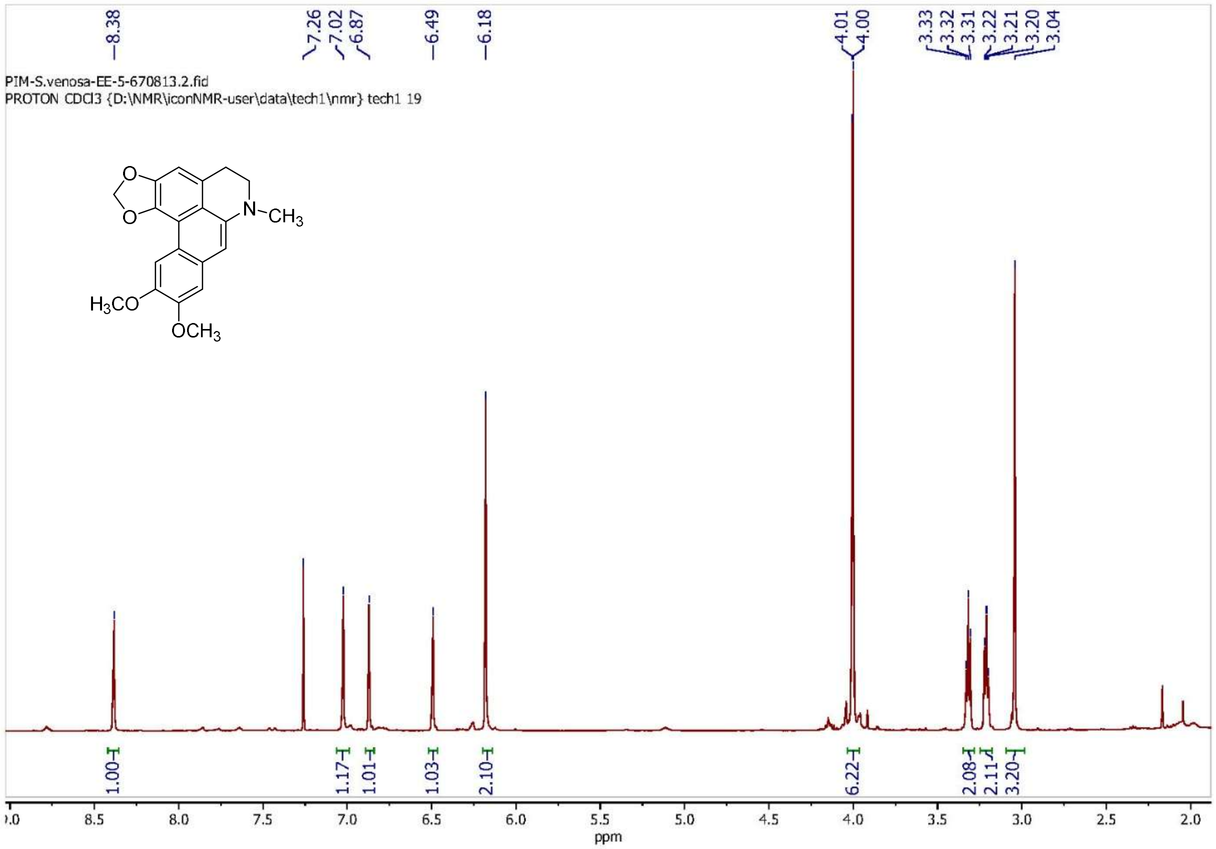

Figure S3. ^{1}H NMR spectrum ($CDCl_3$, 500 MHz) of dehydrodicentrine (**2**)

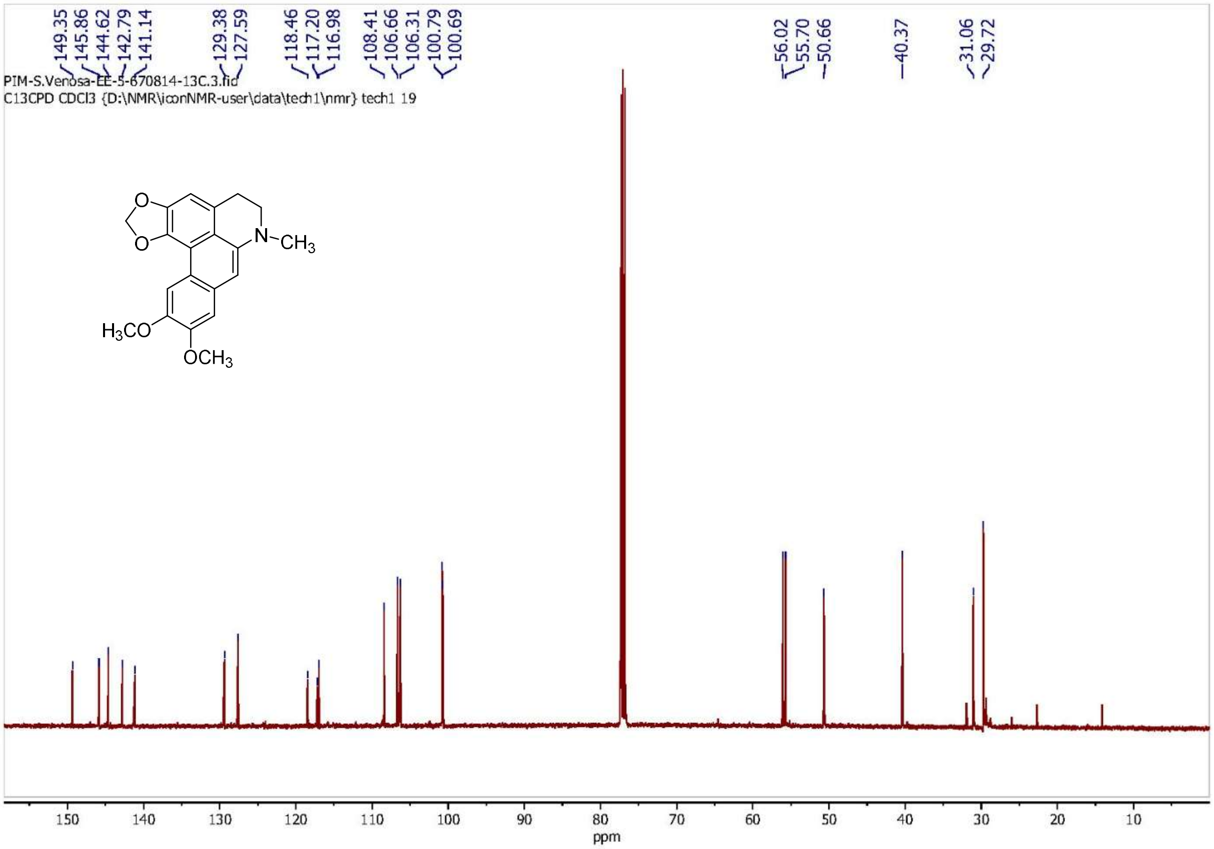

Figure S4. ^{13}C NMR spectrum ($CDCl_3$, 125 MHz) of dehydrodicentrine (**2**)

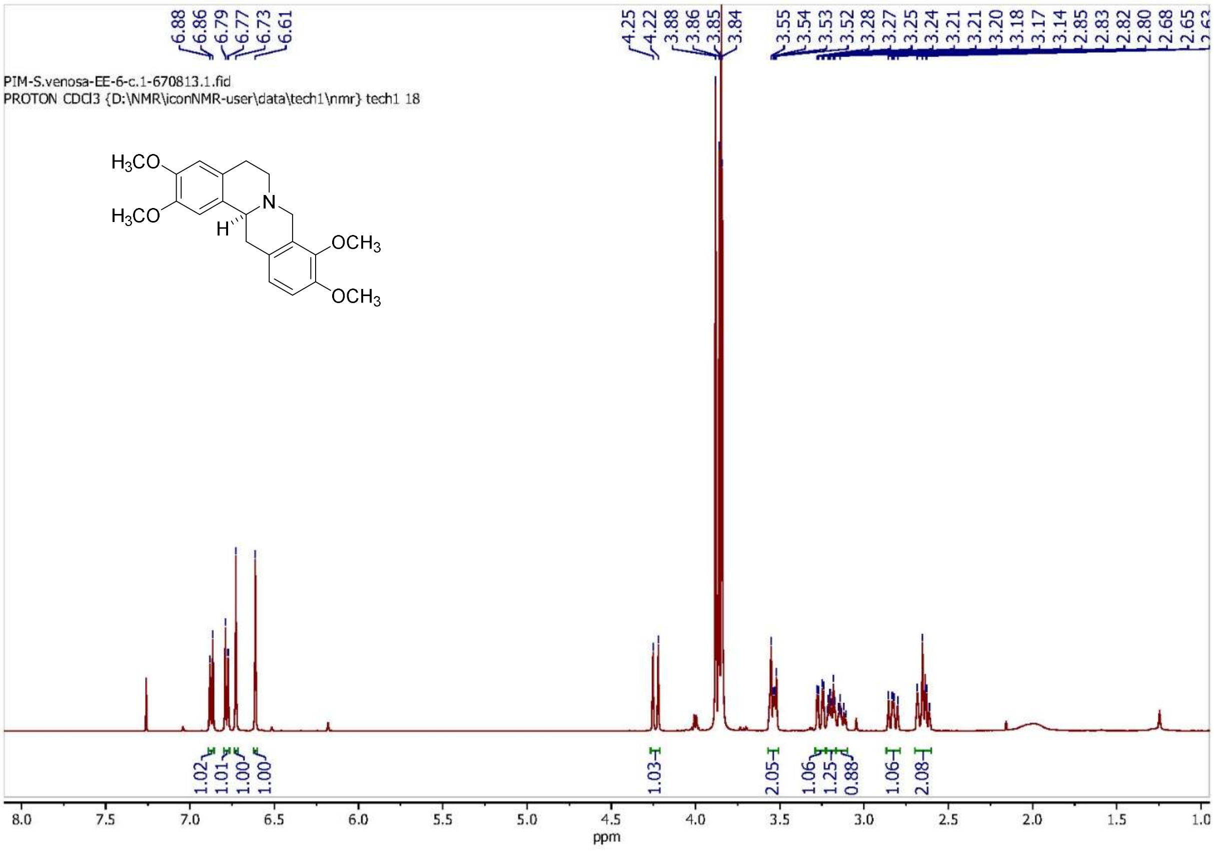

Figure S5. ^{1}H NMR spectrum ($CDCl_3$, 500 MHz) of tetrahydropalmatine (**3**)

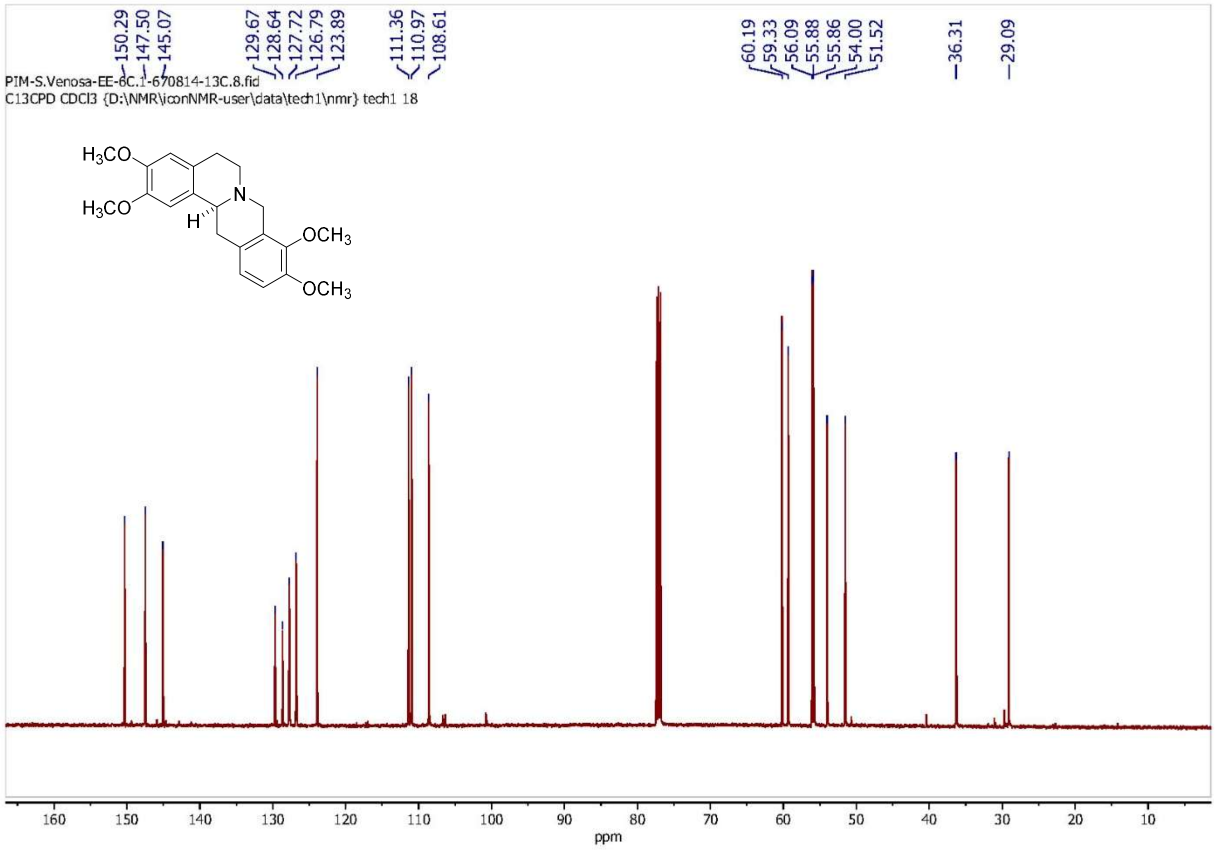

Figure S6. ^{13}C NMR spectrum ($CDCl_3$, 125 MHz) of tetrahydropalmatine (**3**)

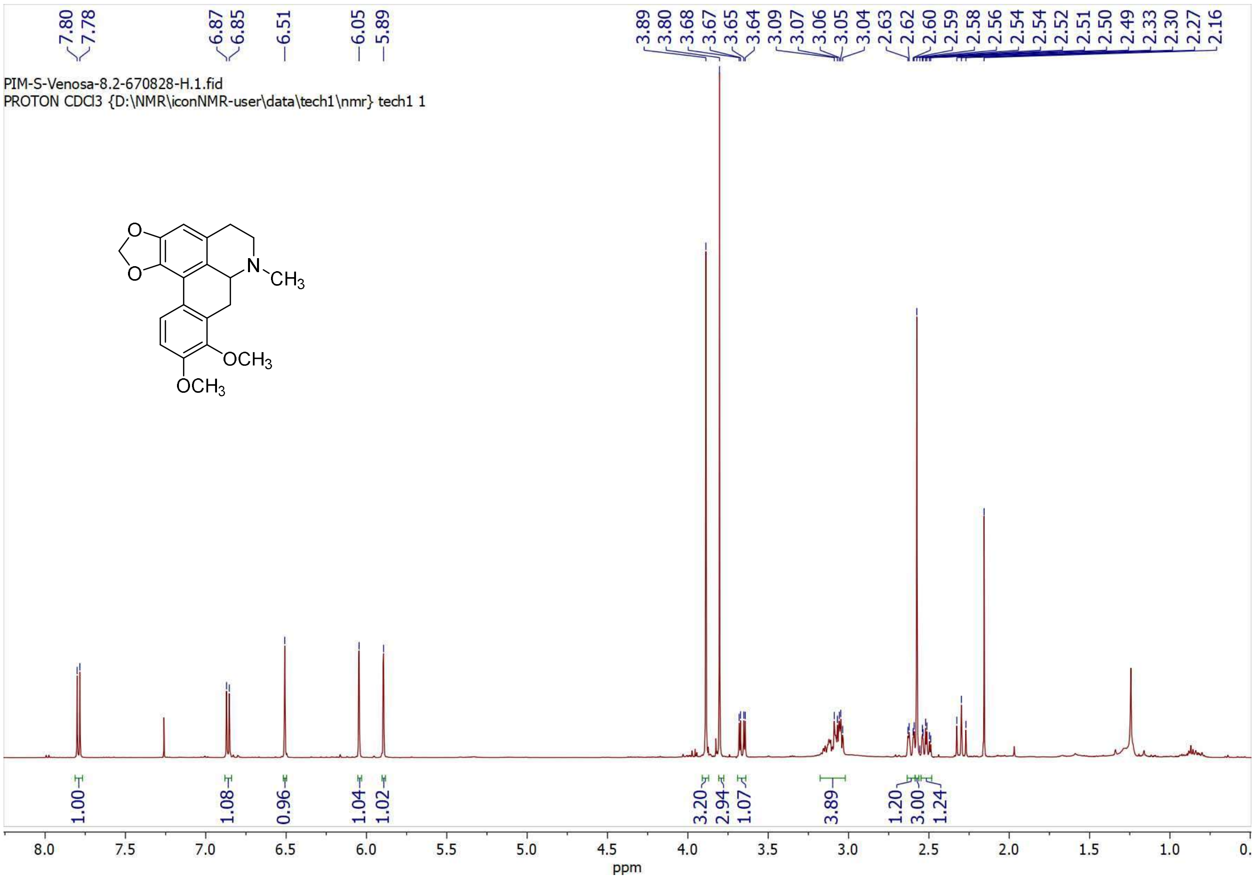

Figure S7. ^{1}H NMR spectrum (CDCl$_3$, 500 MHz) of crebanine (**4**)

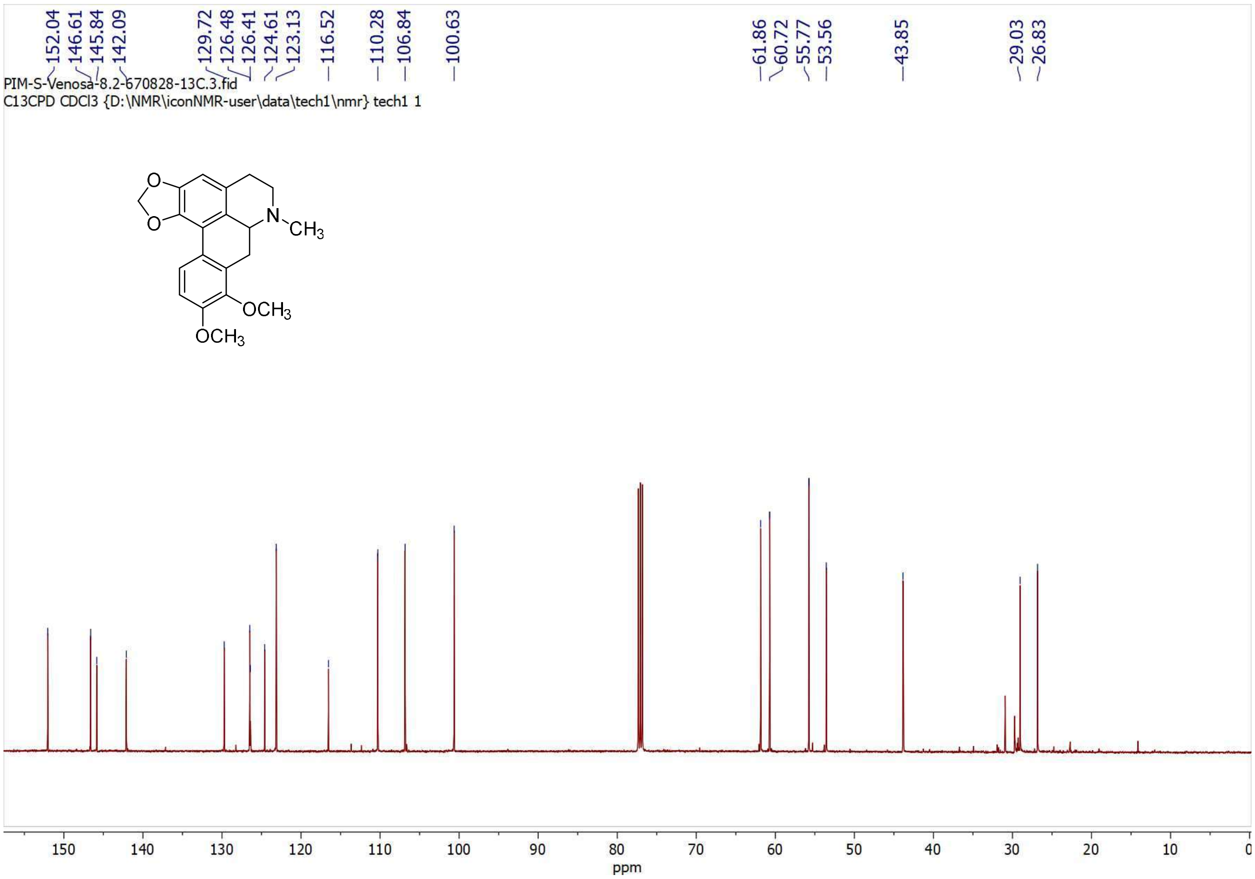

Figure S8. ^{13}C NMR spectrum ($CDCl_3$, 125 MHz) of crebanine (**4**)

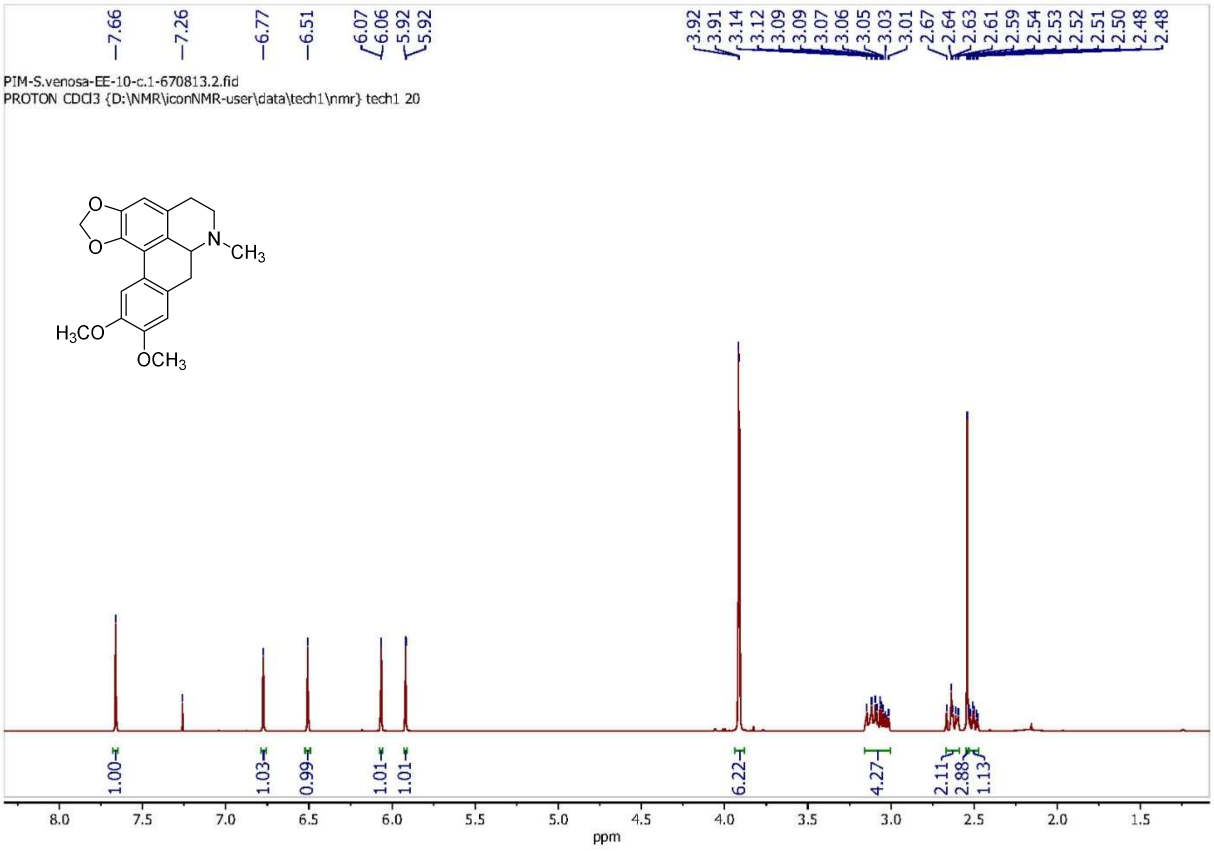

Figure S9. ^{1}H NMR spectrum ($CDCl_3$, 500 MHz) of dicentrine (**5**)

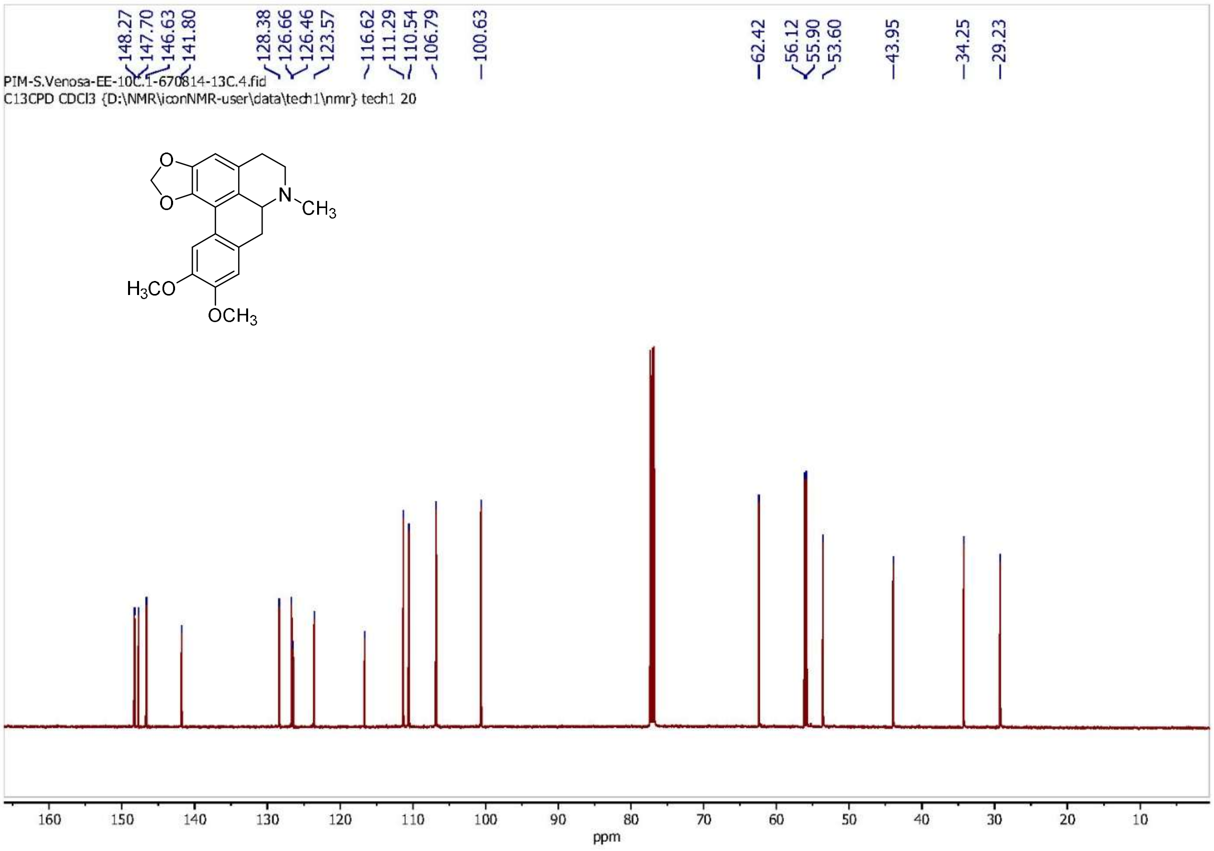

Figure S10. ^{1}H NMR spectrum ($CDCl_3$, 125 MHz) of dicentrine (**5**)

```
File        :C:\msdchem\1\DATA\Aroonchai\SVenosa_210726\SVenosa_EE_2-1_2.D
Operator    : Aroonchai
Acquired    : 21 Jul 2026  10:55     using AcqMethod SVENOSA.M
Instrument  :    GCMS
Sample Name: SVenosa_EE_2-1_2
Misc Info   : 2000 ppm
Vial Number: 3
```

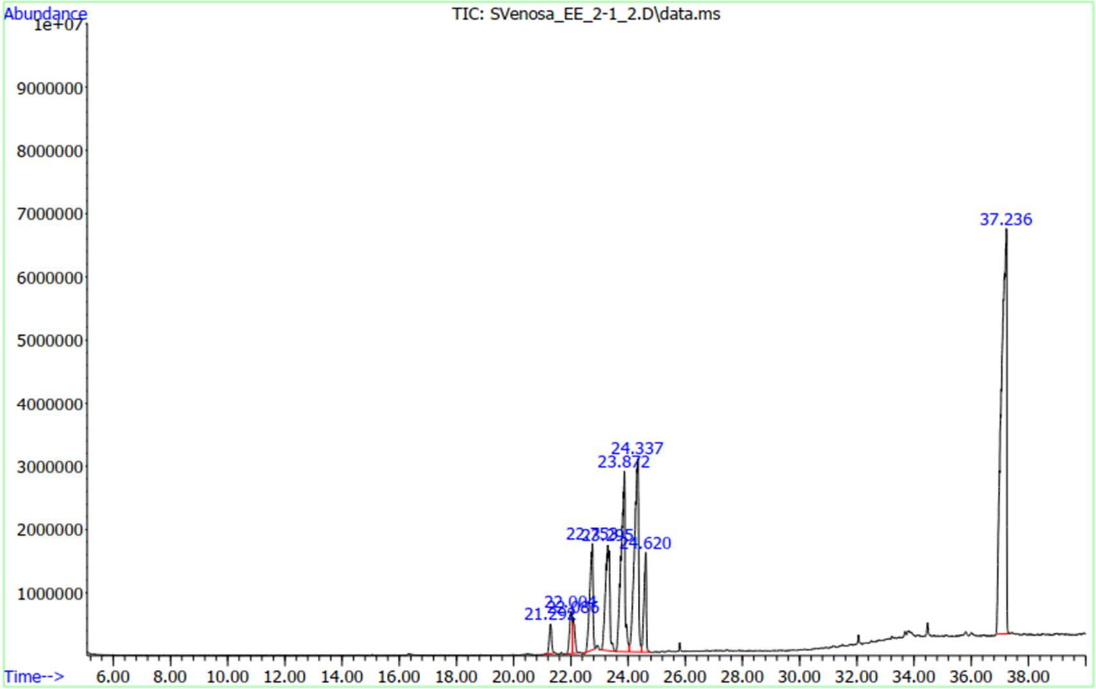

Figure S11. GC chromatogram of compound (**1**)

```
File       :C:\msdchem\1\DATA\Aroonchai\SVenosa_210726\SVenosa_EE_5.D
Operator   : Aroonchai
Acquired   : 21 Jul 2026  10:09     using AcqMethod SVENOSA.M
Instrument :    GCMS
Sample Name: SVenosa_EE_5
Misc Info  : 2000 ppm
Vial Number: 2
```

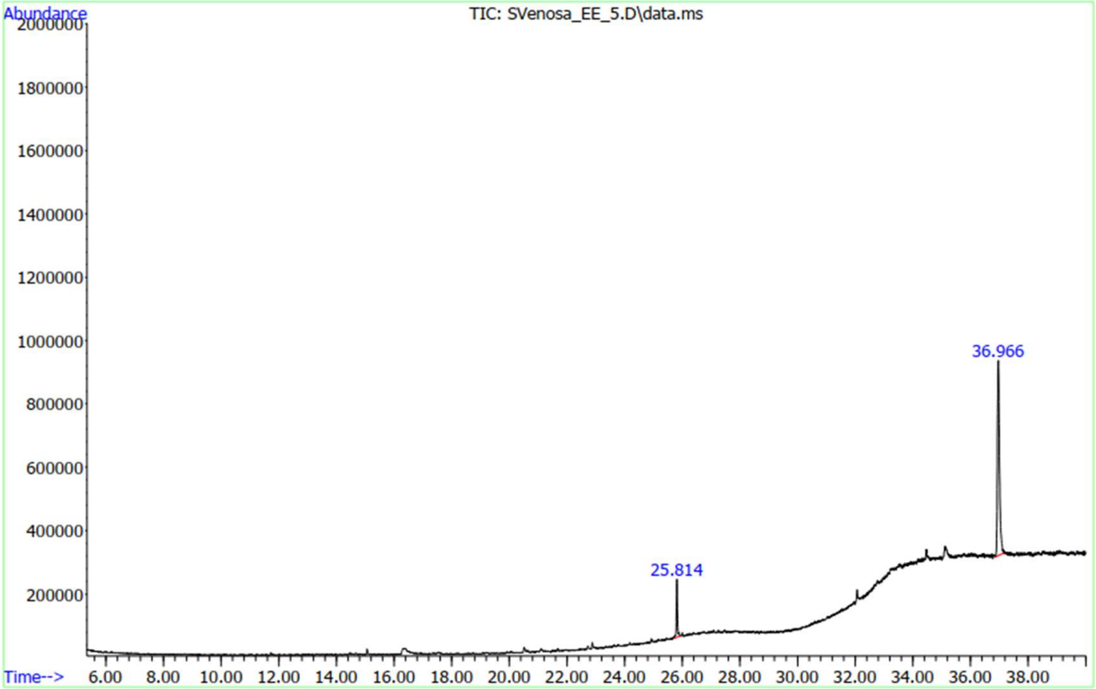

Figure S12. GC chromatogram of compound (**2**)

```
File       :C:\msdchem\1\DATA\Aroonchai\SVenosa_210726\SVenosa 6.D
Operator   : Aroonchai
Acquired   : 21 Jul 2026  13:13     using AcqMethod SVENOSA.M
Instrument :    GCMS
Sample Name: SVenosa 6
Misc Info  : 2000 ppm
Vial Number: 6
```

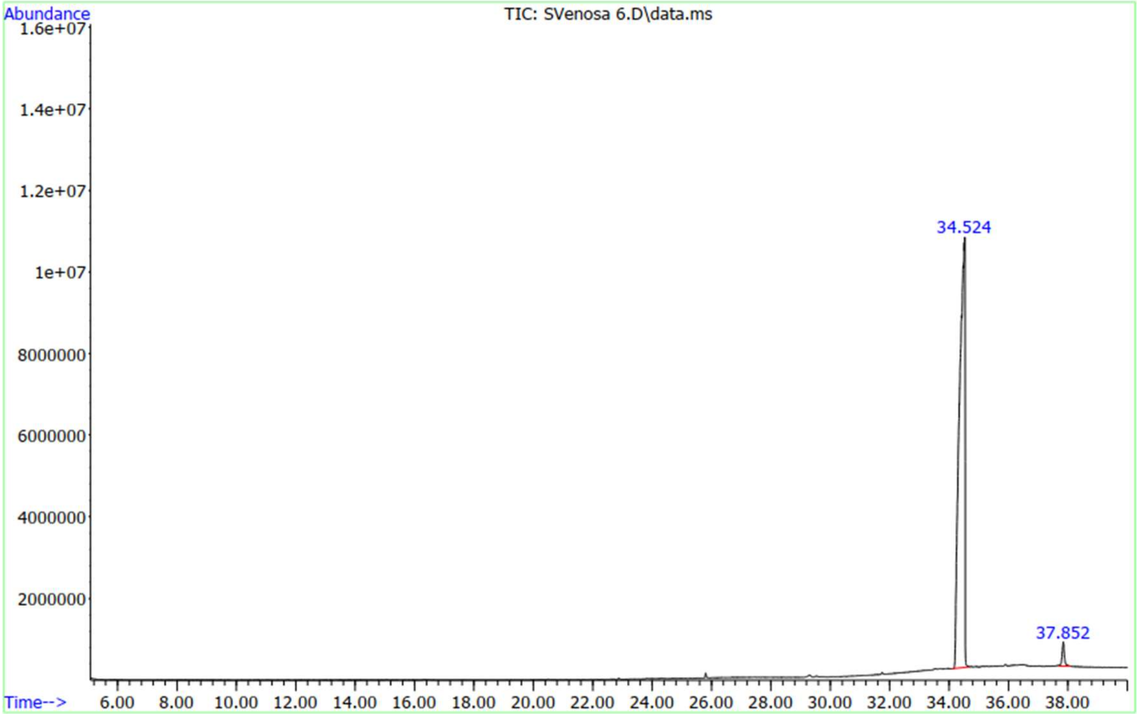

Figure S13. GC chromatogram of compound (**3**)

```
File        :C:\msdchem\1\DATA\Aroonchai\SVenosa_210726\SVenosa 8-2.D
Operator    : Aroonchai
Acquired    : 21 Jul 2026  12:27     using AcqMethod SVENOSA.M
Instrument :    GCMS
Sample Name: SVenosa 8-2
Misc Info   : 2000 ppm
Vial Number: 5
```

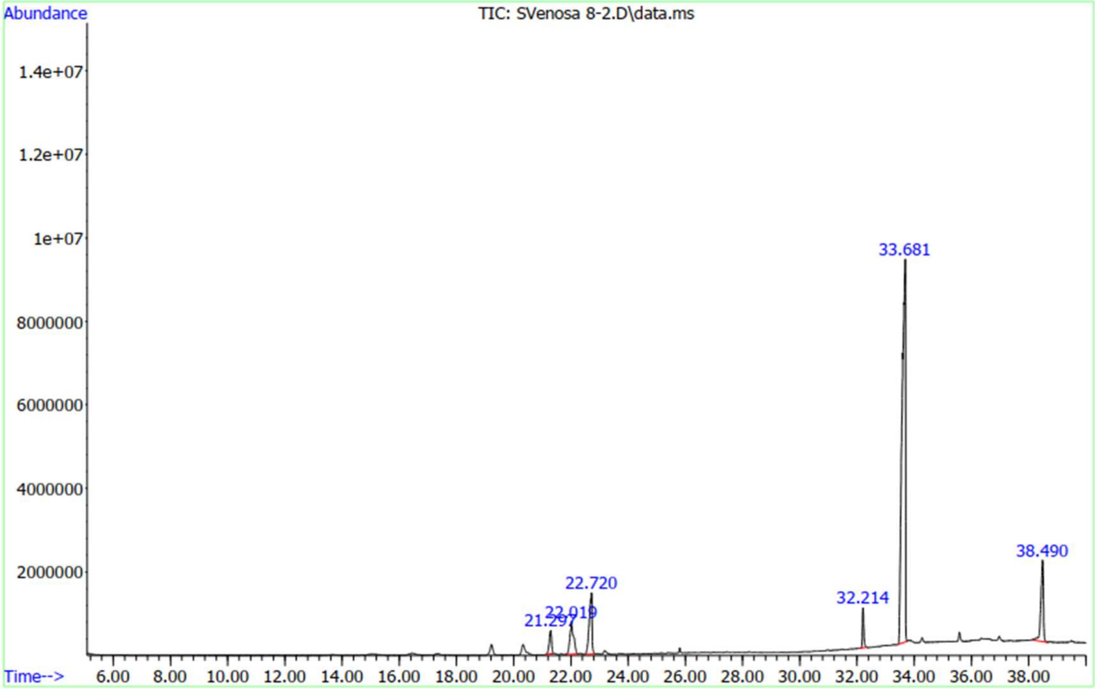

Figure S14. GC chromatogram of compound (**4**)

```
File       :C:\msdchem\1\DATA\Aroonchai\SVenosa_210726\SVenosa 9 10.D
Operator   : Aroonchai
Acquired   : 21 Jul 2026  11:41     using AcqMethod SVENOSA.M
Instrument :    GCMS
Sample Name: SVenosa 9 10
Misc Info  : 5000 ppm
Vial Number: 4
```

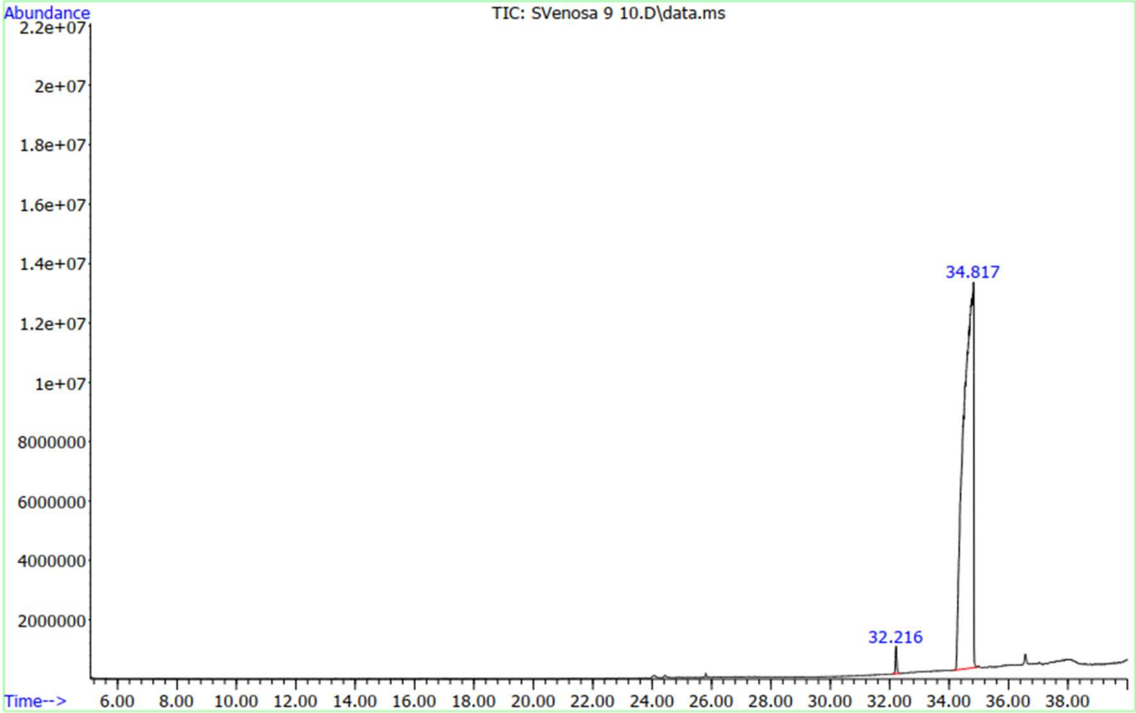

Figure S15. GC chromatogram of compound (**5**)
